# C5orf46: a promising prognosis risk indicator with implication in the remodeling of KIRC and pan-cancer tumor microenvironments

**DOI:** 10.3389/fonc.2026.1713635

**Published:** 2026-05-04

**Authors:** Lei Miao, Fei Wang, Xuzhi Wang, Huijun Yang, Jiayao Li, Siying Liu, Ningning Shen, Zhiqing Yang, Lifang Gao, Wenxia Ma, Chen Wang

**Affiliations:** 1 Basical College of ShanXi Medical University, Taiyuan, Shanxi, China; 2 Second Clinical Medical College of ShanXi Medical University, Taiyuan, Shanxi, China; 3 Public Health School of ShanXi Medical University, Taiyuan, Shanxi, China; 4 Department of Pathology, Second Hospital of ShanXi Medical University, Taiyuan, Shanxi, China

**Keywords:** C5orf46, clear cell renal cell carcinoma, immune infiltration, prognosis risk, tumor microenvironment

## Abstract

**Introduction:**

C5orf46 is a recently discovered tumor-progression-related gene whose function in most cancers is still unknown, especially its potential role in regulating the tumor microenvironment (TME). The aim of the study is to explore the function of the C5orf46 gene in human pan-cancer, including kidney renal clear cell carcinoma (KIRC), for potential clinical application.

**Methods:**

The study started with the physicochemical properties of C5orf46, then the gene expression as well as alteration patterns in diverse cancers, followed by its post-transcription modulation, and then survival analysis. Moreover, the correlations between C5orf46 and multiple cancer TME-related parameters, including angiogenesis, extracellular matrix (ECM) degradation, and immune infiltration, were explored sequentially. Furthermore, C5orf46’s association with other critical cancer features, for instance, cancer stemness, tumor epithelial–mesenchymal transition (EMT), and DNA repair, was also investigated.

**Results:**

Firstly, the physicochemical properties, including amino acid composition, estimated molecular weight, and protein half-life, of the C5orf46 gene were computed sequentially. Then, based on gene expression as well as survival analysis results, C5orf46 was shown to be upregulated in various human cancers, wherein KIRC showed the greatest difference in C5orf46 expression between cancer and corresponding normal tissues. The change in expression was partly due to DNA methylation modulation. Meanwhile, of greater clinical significance, the upregulated C5orf46 expression was correlated with both worse patients’ overall survival and shorter recurrence-free survival. Moreover, the association between C5orf46 and multiple critical cancer traits, including microenvironment angiogenesis, immune infiltration, ECM degradation, and cancer EMT, was validated. Furthermore, the C5orf46 gene was indicated to correlate with the sensitivity of several chemotherapy-related drugs.

**Conclusions:**

Based on TCGA pan-cancer data and validation of local hospital samples, C5orf46 was indicated to potentially work as an oncogene in diverse cancers, and the gene was associated with multiple critical cancer traits.

## 1 Introduction

Cancer remains a global public health threat. The initiation and progression of cancer involve not only the accumulation of genetic mutations, epigenetic alterations, and dysregulated expression of oncogenes and tumor suppressor genes but also the intercellular communication and interaction between cancer cells and the surrounding microenvironment (TME) (1). Over the decades, with the deepened understanding of cancer-associated risk factors, especially the identification of various oncogenes, remarkable achievement has been received in the clinical treatment of cancer (2–4). However, cancer is characterized by high complexity and intratumor heterogeneity, which facilitate the rapid emergence of diverse phenotypes associated with tumor progression. As a result, current therapeutic strategies remain far from meeting clinical demands. The continuous identification of novel gene markers and potential therapeutic targets is therefore of great significance in advancing cancer treatment.

Cancer TME is a key factor implicated in the development of various cancers. It has been increasingly recognized as a complex yet crucial ecosystem, encompassing not only cancer cells but also a wide range of non-malignant cells, including immune cells, cancer-associated fibroblasts (CAFs), endothelial cells, and other tissue-specific cell types (5). An increasing number of studies indicate that the interaction between cancer cells and the TME exerts profound effects on the critical clinical characteristics of tumors, such as cell proliferation, cancer metastasis, microenvironment angiogenesis, and significant immune surveillance escape (6, 7). Depending on the primary organ of cancer origin, intrinsic features of cancer cells, tumor grade and stage, patient’s characteristics, cellular composition, and functional status of the TME, considerable heterogeneity is exhibited. Meanwhile, various cells in the TME can exert either tumor-suppressive or tumor-promoting functions (1, 8, 9).

As one of the primary mediators of the crosstalk between cancer cells and stromal cells, exosomes, which are extracellular vesicles with a diameter of 30–100 nm, are secreted by various TME cells; they contain various bioactive molecules such as nucleic acids, proteins, and lipids (10, 11). Exosomes serve as critical vehicles for intercellular communication, forming a signaling network system that mediates the interactions among various TME components. Tumor-cell-derived exosomes (TDEs) have been reported to play pivotal roles in multiple tumorigenic processes, including promoting tumor epithelial–mesenchymal transition (EMT), mediating immune escape, remodeling the TME, inducing angiogenesis, and regulating macrophage polarization (12–14). The diverse functions of exosome has attracted widespread attention in cancer research. Further investigation into the roles of exosomes and their cargo proteins or nucleic acids in cancer is clinically relevant. The results shall provide valuable insights into the collaborative regulatory network of the cancer TME and facilitate the identification of potential cancer biomarkers.

In this study, we focused on C5orf46, a recently identified cancer-related gene. Multiple online databases, including GeneCards and UniProt, predict that C5orf46 localizes to extracellular exosomes. C5orf46, short for chromosome 5 open reading frame 46, has been reported to be associated with the patient survival in gastrointestinal tumor (15) and some renal and gastric cancers (16, 17). However, the full spectrum of C5orf46’s role in human cancers, especially its potential regulation on cancer TME remodeling, for instance, tumor angiogenesis, immune regulation, and extracellular matrix (ECM) degradation, as a predicted exosome-containing gene, remains to be elucidated.

Based on The Cancer Genome Atlas (TCGA) pan-cancer data and clinical cancer samples from local hospital, we comprehensively analyzed the expression pattern and clinical relevance of C5orf46 across human cancers. Kidney renal clear cell carcinoma (KIRC) was indicated to be the cancer with the greatest difference of C5orf46 expression between cancer and corresponding normal tissues. Additionally, although KIRA patients can benefit from PD-1/PD-L1 blockade immunotherapy, the utility of commonly used immune biomarkers, including PD-L1 expression, microsatellite instability (MSI) and tumor mutation burden (TMB), in KIRC remains controversial. Thus, a comprehensive analysis of the cancer immune microenvironment may provide valuable insights to optimize tumor immunotherapy. Therefore, KIRC was a major focus during our validation of the association between C5orf46 and pan-cancer clinical traits. The findings of the study are expected to enhance our understanding of the role of exosomal C5orf46 in human cancers and assist the unearthing of potential novel prognostic indicators.

## 2 Methods

### 2.1 Data source: TCGA pan-cancer gene expression profiles

TCGA pan-cancer gene expression profiles were used as the primary bioinformatic data source. The study initiated with TCGA pan-cancer data analysis, followed by validation using clinical samples from a local hospital. TCGA data were accessed and downloaded via UCSC Xena (18). Based on the retrieved TCGA datasets, the basic characteristics of C5orf46, especially its expression levels across pan-cancers, and its association with cancer clinical parameters, for instance, cancer stage and grade, subtype, patient age, and gender, were sequentially explored.

### 2.2 Basic physicochemical property analysis of the C5orf46 gene

The basic genetic information and physicochemical properties of the C5orf46 gene was systematically investigated based on a combination of five bioinformatic databases, including ProtParam (19), ProtScale (20), Uniprot (21), GeneCards (22), and Human Protein Atlas (23). ProtParam and ProtScale were jointly applied to investigate the physicochemical properties of the protein, including its computed amino acid composition, encoded protein molecular weight, theoretical isoelectric point, estimated protein half-life, instability index as well as hydrophobicity and hydrophilicity. Meanwhile, Uniprot, GeneCards, and Human Protein Atlas were queried to investigate the cellular location of C5orf46 and its expression patterns in human cancers.

### 2.3 Expression in pan-cancer and association with cancer clinical parameters

Following the characterization of C5orf46’s basic genetic and protein properties, UALCAN (24), which has been an effective and open-access online service for interpreting the association between certain genes and cancer clinical parameters, was employed to investigate C5orf46 expression in cancers compared to that in normal control samples.

Firstly, based on UALCAN, C5orf46 expression patterns were analyzed across human pan-cancer, including but not limited to high-morbidity and high-mortality cancers, for instance, breast invasive carcinoma (BRCA), pancreatic adenocarcinoma (PAAD), rectum adenocarcinoma (READ), hepatocellular carcinoma (LIHC), lung adenocarcinoma (LUAD), lung squamous cell carcinoma (LUSC), kidney clear cell carcinoma (KIRC), and stomach adenocarcinoma (STAD), with corresponding normal tissues as controls. Besides that, the association between C5orf46 expression level and critical clinical parameters, including cancer stage and grade and other cancer features, was evaluated in these cancers.

Subsequently, beyond UALCAN online database analysis, local hospital samples were also used to explore the association between C5orf46 expression and cancer clinical features. Considering that KIRC was the cancer with the greatest C5orf46 gene expression difference between cancer and corresponding normal samples, the analysis was mainly focused in KIRC. A total of 39 local hospital KIRC samples were applied for further tissue microarray production and immunohistochemistry (IHC) experiments. Further experiments that were included in the study, for instance, the expression analysis of C5orf46, its association with CD31- and CD34-stained angiogenesis, as well as the CD8+ T cell infiltration analysis, were all conducted using these samples.

### 2.4 Tissue microarray production

KIRC and giant cell tumor of bone (GCTB) samples were used to make tissue microarrays for a further IHC experiment. The cancer tissues were all obtained from a local hospital biobank. The scientific use of the biobank samples in this study was approved by both the Biobank Committee and Hospital Institutional Board (Second Hospital of ShanXi Medical University, China).

A total of 39 KIRC cases and 20 GCTB cases were selected from the biobank after a confirmation of the disease diagnosis and evaluation of cancer percentage by the local hospital’s registered pathologists based on hematoxylin–eosin (H&E) staining. To mitigate the impact of tumor heterogeneity, three to four regions were marked in each sample case, followed by preparing the receptor wax block with a 1.5-mm needle according to operating instructions (Chloe, Beijing, China). Furthermore, the tissue microarray was serially sliced and stored in a refrigerator at 4 °C for the next step of the experiment.

### 2.5 Immunohistochemistry experiments

#### 2.5.1 Tissue samples and reagents

An immunohistochemistry (IHC) experiment was conducted using the abovementioned cancer tissue microarrays to detect the gene’s expression pattern in different cancer samples. It was performed on VENTANA platform (Roche) in the local hospital’s Pathology Department. The primary antibody of the C5orf46 gene was purchased from Invitrogen (PA5-140360), and the secondary antibody (Envision/HRP kit) and DAB detection kit were from ZSBG-Bio. The other reagents, including but not limited to H_2_O_2_, antigen retrieval citrate solution, phosphate-buffered saline (PBS), and hematoxylin stain, were all supplied by our hospital’s Supply Department.

#### 2.5.2 IHC experimental protocol

The tissue microarray slides were processed by deparaffinization, rehydration with gradient ethanol, and followed by antigen retrieval, according to the antibody’s manual instruction. Meanwhile, to inhibit the activity of endogenous peroxidase, the slides were maintained in 0.3% H_2_O_2_ containing methanol for 10 min. Furthermore, the slides were soaked in bovine serum albumin for 30 min and then incubated with primary C5orf46 antibody (dilution 1:200) overnight at 4 °C and followed by 40 min of secondary antibody incubation at 37 °C. Finally, the slides were processed with horseradish peroxidase (HRP) and visualized in DAB for result evaluation.

#### 2.5.3 IHC result evaluation

**Figure 1:**
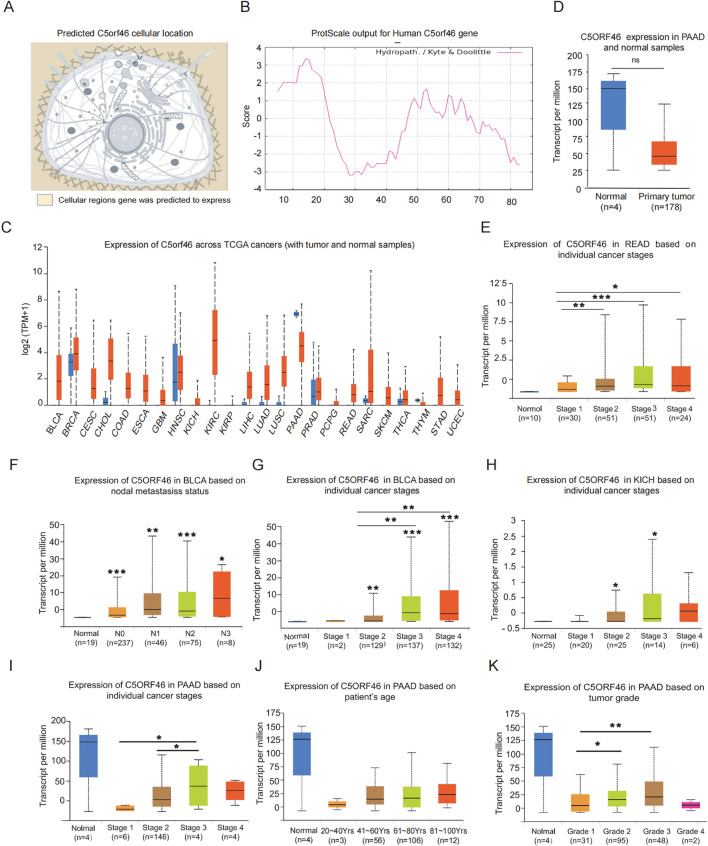
C5orf46 gene characteristics and association with human cancer pathological features. **(A)** Prediction model of the cellular location of C5orf46 gene based on Uniprot platform. **(B)** Computed hydrophilicity/hydrophobicity analysis of C5orf46-encoding protein based on ProtParam and ProtScale database. **(C)** UALCAN prediction of C5orf46 expression in human pan-cancers compared to corresponding normal control samples based on TCGA profiles. **(D)** GEPIA analysis of the C5orf46 gene expression in PAAD compared to normal pancreatic tissues. **(E)** Expression of C5orf46 based on cancer stages in READ. **(F)** Expression of C5orf46 in BLCA based on lymph node metastasis status. Expression of C5orf46 based on cancer stages in **(G)** BLCA and **(H)** KICH. C5orf46 gene expression association with PAAD **(I)** stages, **(J)** patients’ age, and **(K)** tumor grade. (*p < 0.05, **p < 0.01, ***p < 0.001. The first layer—which is right above the error bar—represents comparison to the normal group, and the above layers—which were above the secondary line represent the comparison between the corresponding groups that were covered by the line.

C5orf46 was observed to distribute mostly in the cell cytoplasm, cell membrane, and extracellular regions. The evaluation of the IHC result was based on both staining intensity and staining area. In the study, the IHC result was evaluated by two registered pathologists of the local hospital, with the staining intensity scored as follows: none (0), mild (1), moderate (2), and strong (3). Meanwhile, the staining area was classified as follows: 10% (0), 11%–25% (1), 26%–50% (2), 51%–75% (3), and >75% (4) (example in **Figures 1A–D**).

The section’s final score equals the product of staining intensity and staining area, and the final result of each patient’s tissue was recorded as the average of three independent microarray cores’ scores, and if the final score <6, the result was defined as low expression. Meanwhile, if the final score ≥6, the result was classified as high expression.

### 2.6 Survival analysis of C5orf46 gene in pan-cancer

Survival analysis was conducted using the Kaplan–Meier plotter (25), which has been an effective database for investigating the correlation of human genes with overall survival (OS) as well as recurrence-free survival (RFS). In the database, over 10,000 cases covering broad-spectrum human cancers were contained. The prognostic value of C5orf46 in pan-cancer was analyzed based on this platform.

### 2.7 Methylation regulation and genetic alteration analysis

To preliminarily explore the potential mechanisms underlying the altered expression of C5orf46 in cancer tissues relative to corresponding normal tissues, the common gene expression regulatory events, particularly methylation and phosphorylation regulations of the gene, were preliminarily explored based on the UALCAN platform, which has been previously used to analyze the association between the gene and the clinical parameters. Based on UALCAN, the relative methylation level of C5orf46 in cancers, as well as the relative phosphorylated protein compared to the total C5orf46 protein levels in cancers, was assessed.

Furthermore, in addition to dysregulated mRNA expression, other types of genetic variations, for instance, gene mutation, copy number variation, amplification, and deletion, are also important genetic changes that may affect the cancers’ biological functions. cBioPortal (26) serves as a comprehensive resource for investigating gene variations in cancer research worldwide. In the study, cBioPortal was applied to firstly uncover the genetic alterations of C5orf46 in human cancers using the “cancer types summary” module of the “quick search” section in the database and then visualize the mutational sites of C5orf46 in 3D protein structures using the “mutation” module of the database.

### 2.8 C5orf46 centering protein–protein interaction network construction and related genes’ analysis

Following the characterization of C5orf46 expression patterns and genetic alterations in cancers, subsequent analyses were performed to elucidate the detailed biological functions of C5orf46 in cancers and preliminarily explore the potential mechanism.

Firstly, STRING, which is short for Search Tool for the Retrieval of Interacting Genes (27), was used to construct the PPI network centering on the C5orf46 gene to investigate its surrounding interacting gene partners. Gene enrichment analysis was then applied to annotate the basic biological attributes of the PPI network containing genes, including their main cellular location, involved biological processes, molecular functions, and the signaling pathways that the genes were mainly enriched in. These analyses provided a preliminary understanding of C5orf46, its potential interacting partners, and their biological features.

### 2.9 TME angiogenesis association of C5orf46 gene in pan-cancer

To validate the association between C5orf46 gene and tumor angiogenesis, 32 well-known genes that were previously reported to be related with TME angiogenesis (listed in **Supplementary Table 1**), including vascular endothelial growth factor (VEGF), fibroblast growth factor (FGF), and platelet-derived growth factor (PDGF), were selected. Furthermore, based on TCGA pan-cancer data, the expression levels of the 32 genes were extracted and uploaded to GEPIA2.0 to calculate their expression correlations with C5orf46.

Subsequently, an IHC experiment was conducted to verify the association between C5orf46 expression and angiogenesis in KIRC tissues. CD31 and CD34, which are two commonly used markers for immature and mature tissue blood vessels, were used during the analysis. Primary antibodies to both CD31 and CD34 were purchased by the hospital’s Pathological Department from ZSGB-BIO (Beijing, China), and the other IHC experiment reagents and protocols were consistent with those described previously. Meanwhile, the Weidner counting method was used to analyze the microvessel density (MVD) in the tissues. A single endothelial cell or an independent, non−connected endothelial cell cluster was regarded as one microvessel. Five areas in the tumor center were randomly selected under a ×100 field of view. Image analysis was then performed under a ×200 field of view, and the mean value was calculated using Image−Pro Plus 6.0 software (as seen in the schematic diagram in **Supplementary Figure 1**).

### 2.10 Extracellular matrix degradation association analysis

In addition to TME angiogenesis, extracellular matrix (ECM) degradation constitutes another major aspect of TME remodeling, which is critically implicated in multiple cancer progression events, most significantly cancer invasion and metastasis. To evaluate the association between C5orf46 and cancer ECM degradation, 23 genes that have been supported to be related with ECM degradation were selected (listed in **Supplementary Table 2**), and based on TCGA pan-cancer expression data, the correlation between C5orf46 and the 23-gene signature was evaluated across various cancer types to investigate the potential role of C5orf46 in TME remodeling.

### 2.11 Association between C5orf46 and cancers’ EMT process

Epithelial–mesenchymal transition (EMT) refers to the transformation of epithelial cells to mesenchymal cells, which endows cells with the ability to transfer and invade, thus promoting multiple critical cancer processes, for instance, inducing stem cell characteristics, reducing apoptosis, and increasing immune suppression. To evaluate the effect that the C5orf46 gene has on cancer EMT process, 14 characteristic genes (listed in **Supplementary Table 3**) that were indicated by previous reports to be EMT-related were identified, and their expression levels were retrieved from TCGA pan-cancer datasets. Furthermore, the correlation between C5orf46 and the 14-gene signature was systematically analyzed.

### 2.12 C5orf46 correlation with cancers’ stemness index

Cancer stem cells represent a distinct cell population exhibiting biological characteristics similar to regular stem cells, which possess high self-renewal capacity and the potential to differentiate into diverse cancer cell lineages. Over the past decade, researchers engaged in TCGA projects analyzed thousands of cells at different stages of cell differentiation, thereby identifying the typical molecular characteristics possessed by stem cells. Based on these data, they developed the “cancer stemness index”, which was calculated based on the genes’ expression and DNA methylation characteristics (28).

The cancer stemness index ranges from 0 to 1, with 0 indicating a low similarity to stem cells and 1 indicating a high similarity. A higher index is closely related with the progression of cancer. In the study, the stemness index was computed across human cancers using the published algorithm, and differences between C5orf46-high- and C5orf46-low-expression subgroups were compared.

### 2.13 C5orf46 correlation with cancers’ HRR signature and MRR proteins’ expression

Given that homologous recombination deficiency (HRD) has emerged as an important clinical feature which correlates not only with disease progression but also with the efficacy of PARPi-related drug therapies (29), we further evaluated the association between C5orf46 and cancer DNA repair ability which was based on TCCA pan-cancer data of 27 selected homologous recombination repair (HRR) signaling-related genes (listed in **Supplementary Table 4**).

In addition to HRD, the potential correlation between C5orf46 expression and another well-known DNA deficiency repair system, namely, mismatch repair (MMR) protein expression, was also analyzed. Four canonical MMR proteins, namely, MLH1, PMS2, MSH2, and MSH6, were detected among the local hospital’s cancer samples. Since deficient mismatch repair (dMMR) is rare in KIRC, the validation was conducted using COAD cancer samples which exhibit a high prevalence of dMMR. The primary antibody to all of the four MMR proteins was purchased by the local hospital’s Pathology Department from ZSGB-BIO (Beijing, China), and the IHC reagents and experiment procedures of the four MMR proteins’ detection were consistent with those described earlier. Furthermore, the C5orf46 expression in COAD samples was detected using qRT-PCR experiment as previously described, and the association between relative C5orf46 expression and MMR status was evaluated by integrating the qRT-PCR and IHC experiment results.

### 2.14 Immune infiltration cells and CTL dysfunction correlation

Beyond EMT, angiogenesis, and ECM degradation, exosomes have also been known to modulate the immune microenvironment. To characterize the immune landscape between C5orf46-high and low-expression cancer samples, CIBERSORT algorithm was performed to calculate the relative contents of 22 tumor-infiltrating immune cells (TICs) based on the TCGA profile data, followed by investigating the correlation between C5orf46 expression and the TICs’ infiltration level.

Notably, cytotoxic T lymphocytes (CTLs) have been a key component of the adaptive immune system and play important roles in human anti-tumor immunity, and CTLs are also one of the main effector cells of tumor immunotherapy. In the study, the association between C5orf46 expression and CTLs’ function on patient survival rates was especially presented based on the tumor immune dysfunction and exclusion (TIDE) platform (30), which has been an open-access and user-friendly platform. By inputting the gene symbol and selecting the corresponding cancer type, CTL-related functional and survival statistics will be automatically generated.

Furthermore, considering that CD8+ T cells represent a major cytotoxic antitumor immune subset, we validated the association between C5orf46 expression and CD8+ T cell infiltration using the same KIRC tissue microarray which has been previously used for CD31 and CD34 staining. The primary antibody to CD8 was purchased from ZSGB-BIO (Beijing, China). Meanwhile, the other IHC reagents, equipment, and experiment procedure were the same as previously described. The evaluation of CD8+ T cells’ infiltration was scored as the average number of positively stained cell numbers in five five randomly selected fields under ×200 magnification.

### 2.15 Tumor mutation burden, microsatellite instability, and immune therapy sensitivity analysis

Microsatellite instability (MSI) and tumor mutation burden (TMB) are currently effectively used as clinical biomarkers to predict the potential benefit of patients from immune therapies. Therefore, following the immune cell infiltration and CTL function analyses, the correlation between C5orf46 expression and pan-cancer TMB and MSI scores was evaluated based on the ACLBI online database (31).

Moreover, based on the ROC plotter analysis platform, the C5orf46 gene expression differences between the responders and non-responders to specific immune therapy drugs, for instance, anti PD-1, PD-L1, and CTLA4 inhibitors, were displayed, along with the receiver operating characteristic curves (ROC) for survival analysis in therapy-related cancer patients.

### 2.16 Chemotherapy-related drugs’ sensitivity analysis

Besides immune therapy, to preliminarily investigate the effects that C5orf46 has on the routine chemotherapeutic response for human cancers, based on ROC plotter analysis platform, the C5orf46 gene expression between the responders and non-responders to commonly used chemotherapy drugs in different types of human cancers, as well as the ROC curve for therapy-related patient survival, was additionally displayed (32).

### 2.17 Statistical analysis

Most of the bioinformatics analyses were performed on corresponding online databases. As for the processing of downloaded TCGA pan-cancer data, statistical analysis was performed using SPSS 26.0. For enumeration data, for instance, when comparing the gene expression difference between cancers and corresponding normal samples, the data were analyzed using *t*-test. As for the measurement data including the association between gene expression and the cancers’ pathological parameters, the data were analyzed by using *χ*^2^-test. For the correlation analysis, for instance, the correlation between C5orf46 gene expression and angiogenesis signature, the data were analyzed by using Spearman analysis. *p* < 0.05 was considered statistically significant (for all analysis results, *p < 0.05, **p < 0.01, ***p < 0.001).

## 3 Results

### 3.1 C5orf46 genetic information and physicochemical property

C5orf46, which is short for chromosome 5 open reading frame 46, is also known as AP-46 and SSSP1. Based on the combined analysis of different platforms, the basic genetic information and the physiochemical properties of the gene were discovered. The results revealed that the C5orf46 gene locates in 5q32, comprises five exons, and encodes a protein containing 87 amino acids. The protein’s formula is C_433_H_686_N_108_O_135_S_4_, with an estimated weight of 9.7 kDa, and the computed theoretical isoelectric point of the protein is 4.67. Meanwhile, the instability index of the protein is estimated to be 39.96, and the grand average of the hydrophobic value is -0.254, indicating that C5orf46 functions as a cellularly stable and hydrophilic protein.

Meanwhile, as for the cellular location, C5orf46 was supported by multiple databases, including Uniprot, GeneCards, and Human Protein Atlas, to be secreted by cells and located in extracellular exosomes (**Figure 1A**), which is consistent with its computed protein stability and hydrophilic property (**Figure 1B**).

### 3.2 C5orf46 was aberrantly upregulated in KIRC and multiple other cancers and was associated with advanced cancer grade and stage

**Figure 2:**
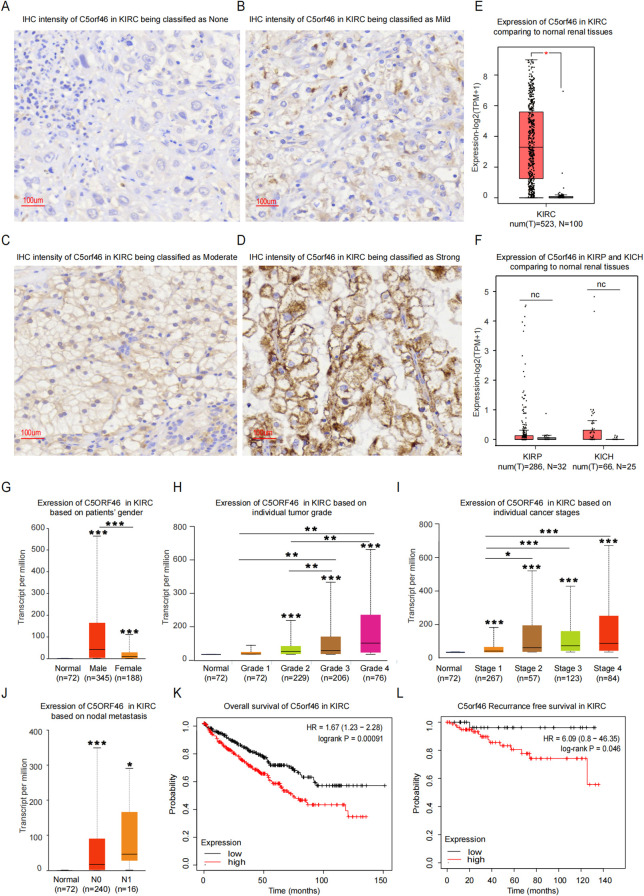
C5orf46 gene expression in KIRC and its association with cancer clinical pathological features. IHC experiment staining of C5orf46 expression in KIRC samples, the intensity of which was scored as **(A)** “none”, **(B)** “mild”, **(C)** “moderate”, and **(D)** “strong” (all figures’ magnification: ×20: the error bar represents 100 um). GEPIA analysis of C5orf46 gene expression in **(E)** KIRC, **(F)** KIRP and KICH compared to corresponding normal tissues. Expression of C5orf46 in KIRC based on **(G)** patients’ gender, **(H)** tumor grade, **(I)** cancer stages, and **(J)** nodal metastasis status (*p < 0.05, **p < 0.01, ***p < 0.001). The first layer—which is above the error bar—represents comparison to the normal group, and the above layers—which were above a secondary line—represent the comparison between corresponding groups that were covered by the line. **(K)** Overall survival and **(L)** recurrence-free survival analysis of C5orf46 in KIRC.

The expression of the C5orf46 gene in broad-spectrum cancers and corresponding normal control samples was investigated, and the results indicated that C5orf46 was statistically significantly upregulated in various cancer types except for PAAD (**Figure 1C**). In PAAD, the gene seemed to be expressed at lower levels in cancer compared with normal samples even though the difference was not statistically significant (**Figure 1D**). Among the upregulated cancers, KIRC tended to be the cancer type with the highest expression difference between cancer and corresponding normal tissues, and the tendency was also validated using cancer samples from a local hospital based on an IHC experiment (**Figures 2A–D**). Although the gene expression was also aberrantly upregulated in other cancers, the difference was not as high as in KIRC, including in other kidney cancers, for instance, KIRP and KICH (**Figures 2E, F**).

Meanwhile, as for the association with KIRC clinicopathological features, C5orf46 was indicated by the TCGA data to tend to be expressed higher in male patients than in female ones (**Figure 2G**), a phenomenon which was discovered in both TCGA and local hospital samples (**Table 1**). The upregulated C5orf46 expression was associated with a more advanced cancer grade and stage (**Figures 2H, I**). Moreover, gene expression was also higher in cases with nodal metastasis, although the difference was not statistically significant, which might be due to the limited number of cases in the metastatic group (**Figure 2J**). The association between C5orf46 expression and KIRC clinical features was also validated using samples from the local hospital, which also revealed a similar trend between C5orf46 expression and the patients’ ISUP grade as well as bone metastasis (the main metastasis site for local KIRC samples). Considering that C5orf46 was indicated to be expressed differently in male and female patients, a stratified analysis based on patients’ gender was additionally conducted, which also indicated that a higher C5orf46 expression was associated with more advanced cancer stages (**Supplementary Table 5**). Interestingly, gene expression was also related with KIRC ISUP grade in female, but not male, patients. A further deeper analysis based on patients’ gender would be of potential clinical value. Meanwhile, no statistically significant correlation was observed between C5orf46 and the patients’ age, tumor size, membrane and renal sinus invasion, and other clinical features.

**Table 1:** Association between C5orf46 expression and KIRC clinical features.

Parameters		C5orf46 expression		*P*-value
		Low expression	High expression	
Gender				
	Male	8 (33.3%)	16 (66.7%)	0.042*
	Female	10 (66.7%)	5 (33.3%)
Age				
	≤40	3 (60.0%)	2 (40.0%)	0.647
	>40	15 (44.1%)	19 (55.9%)
WHO/ISUP grade				
	G1/G2	14 (60.9%)	9 (39.1%)	0.027*
	G3/G4	4 (25.0%)	12 (75.0%)
Tumor diameter				
	≤4 cm	6 (54.5%)	5 (45.5%)	0.883
	4–7 cm	10 (41.7%)	14 (58.3%)	
	>7 cm	2 (50.0%)	2 (50.0%)	
Membrane invasion				
	Yes	14 (48.3%)	15 (51.7%)	0.932
	No	4 (40.0%)	6 (60.0%)
Neurovascular invasion				
	Yes	1 (33.3%)	2 (66.7%)	0.643
No	17 (47.2%)	19 (52.8)
Renal sinus invasion				
	Yes	2 (33.3%)	4 (66.7%)	0.497
	No	16 (48.5%)	17 (51.5%)
Renal pelvis invasion				
	Yes	4 (80.0%)	1 (20.0%)	0.162
	No	14 (41.2%)	20 (58.8%)
T stage				
	Ia	5 (50.0%)	5 (50.0%)	0.883
	Ib	10 (63.6%)	14 (36.4%)	
	II	2 (50.0%)	2 (50.0%)	
Bone metastasis				
	Yes	4 (23.5%)	13 (76.5%)	0.013*
	No	14 (63.6%)	8 (36.4%)

*p < 0.05.

As for the other cancers, a similar association has also been discovered between the C5orf46 gene and the clinical features in multiple cancers, for instance, a higher gene expression was related with more advanced cancer stages in READ, BLCA, and KICH (**Figures 1E, G, H**), and it was also expressed higher in cases with nodal metastasis in BLCA (**Figure 1F**). Interestingly, similar trends were observed between C5orf46 and PAAD clinical features, which was revealed by previous analysis to be the only cancer with downregulated C5orf46 expression. C5orf46 expression was also higher in more advanced PAAD stages and grades. Even though the expression in grade 4 and stage 4 seemed to be lower than grade 3 and stage 3 (**Figures 1I–K**), the difference was not statistically significant, and it might be the result of the limited sample cases in the fourth group. A similar trend in cancer stages and grades in PAAD, as observed in KIRC and other cancers, indicates that C5orf46 functions as an oncogene in these cancers.

### 3.3 Higher expression of C5orf46 correlated with worse patient survival in cancers

**Figure 3:**
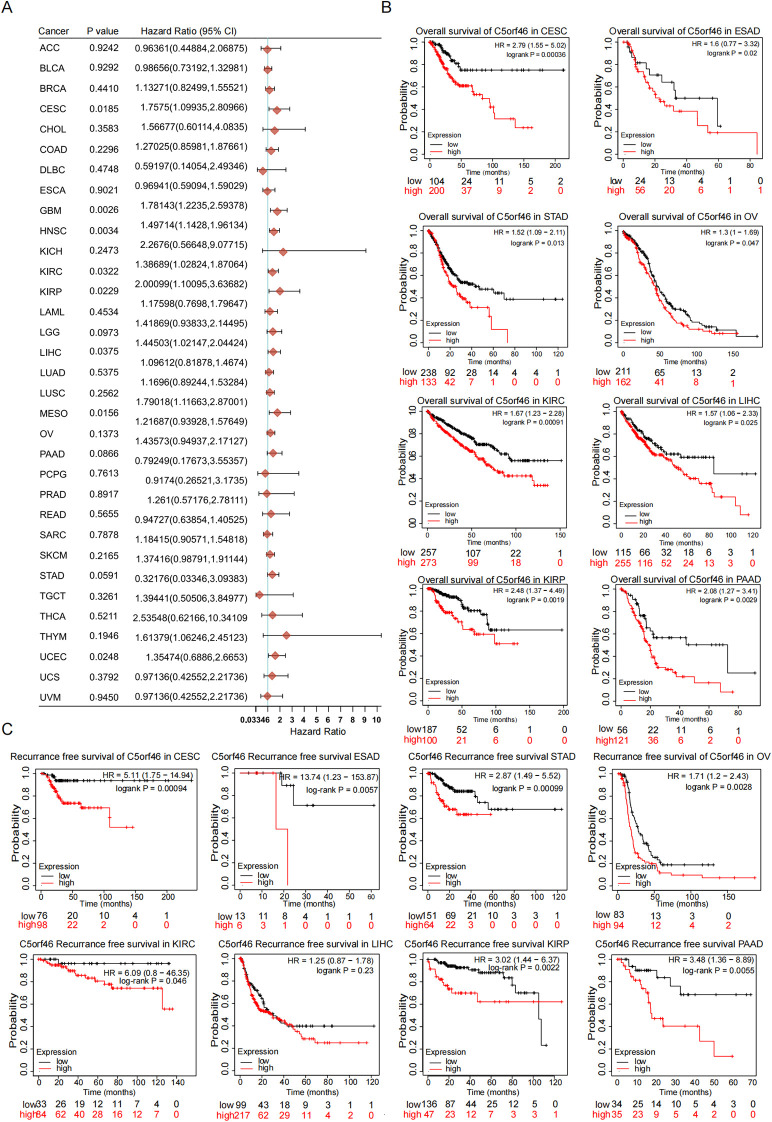
Association between C5orf46 gene expression and pan-cancer patient survival. **(A)** C5orf46 gene association with the patient survival risk in pan-cancer. **(B)** Association between C5orf46 and the patients’ overall survival in different types of cancers. **(C)** Association between C5orf46 and the patients’ recurrence-free survival in different types of cancers (*p* < 0.05 was considered statistical significant).

Besides the association with advanced cancer grades and stages, the direct prognosis indicating the value of the C5orf46 gene was analyzed in the next step. The results indicated that a higher expression of the gene was associated with statistically significantly worse overall survival (OS) and shorter recurrence-free survival (RFS) in nearly all cancers, including KIRC (**Figures 2K, L**) and others—for instance, CESC, STAD, PAAD, and KIRP (**Figures 3A–C**). The consistent results showing the association with both advancing cancer stages and worse patient survival, including in PAAD, supported a previous speculation that C5orf46 functions as a potential oncogene in a broad spectrum of human cancers.

### 3.4 C5orf46 was correlated with cancer metastasis status

**Figure 4:**
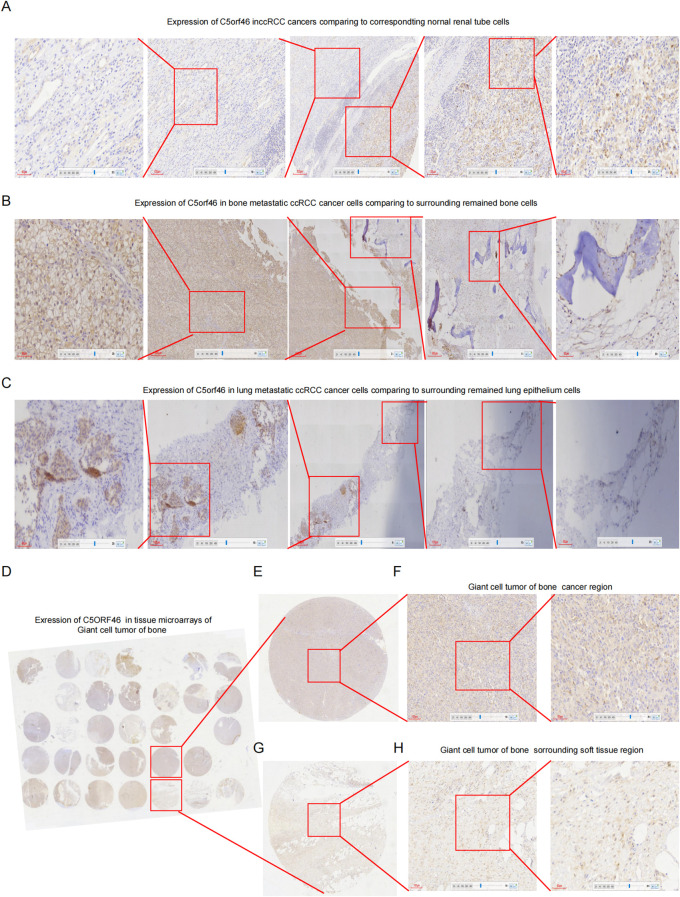
IHC detection of C5orf46 expression in primary and metastatic KIRC samples. **(A)** Expression of C5orf46 in KIRC cancers compared to corresponding normal kidney cells. The middle graph in the panel reflects cancer and the surrounding normal tissues; magnification: ×40; the error bar represents 300 um. The nearer left and right graphs represent normal kidney and cancer lesion, respectively: magnification: ×100; the error bar represents 100 um. The further left and right graphs represent the magnification of the nearer graph; magnification: ×200; the error bar represents 60 um. **(B)** Expression of C5orf46 in bone metastatic KIRC cancers (the middle graph contains cancer and the surrounding remaining bone tissues; magnification: ×40; the error bar represents 300 um). The nearer and further left and right graphs represent cancer and the surrounding remaining bone tissues; magnification: ×100 and ×200; the error bar represents 100 and 60 um, respectively. **(C)** Expression of C5orf46 in lung metastatic renal cancers (the middle graph contains cancer and the surrounding remaining lung tissues; magnification: ×40; the error bar represents 300 um). The nearer and further left and right graphs represent cancer and the surrounding remaining lung tissues; magnification: ×100 and ×200; the error bars represent 100 and 60 um, respectively. **(D)** A tissue microarray made of giant cell tumor of bone samples for preliminary detection of C5orf46 expression in soft-tissue-originating tumors. **(E)** Magnification of a tumor tissue based on **(D)** microarray, and IHC experiment was conducted on it. **(F)** IHC experiment detection of C5orf46 expression in the case of a giant cell tumor of the bone (×magnification: ×100 and ×200; the error bar represents 100 and 60 um in the left and right graph, respectively). **(G)** Magnification of the surrounding normal soft tissue based on **(D)** microarray, and IHC experiment was conducted on it. **(H)** IHC experiment detection of C5orf46 expression in the surrounding normal soft tissues (magnification: ×100 and ×200; the error bar represents 100 and 60 um in the left and right graph, respectively).

Cancer metastasis has been a major reason for poor patient prognosis, and to evaluate the association between C5orf46 and cancer metastasis, an IHC experiment was applied on KIRC, including other cancer samples. The results revealed that C5orf46 expression was higher not only in KIRC cancer cells compared with corresponding normal kidney cells (**Figure 4A**) but also in bone (**Figure 4B**) and lung metastatic (**Figure 4C**) KIRC cancer cells compared with the surrounding remaining normal bone and lung tissues. Meanwhile, besides epithelium-originated cancers, for the preliminary detection of C5orf46 expression in mesenchymal-tissue-originated tumors, a tissue microarray made of GCTB was also applied to detect C5orf46 expression (**Figure 4D**), and the results revealed that the gene expression was higher in tumor lesions (**Figures 4E, F**) compared with the surrounding normal soft tissues (**Figures 4G, H**), although the gene seemed to be diffusely distributed with no difference in tumor or immune cells.

### 3.5 Altered C5orf46 expression in cancers was partly attributed to DNA methylation regulation

**Figure 5:**
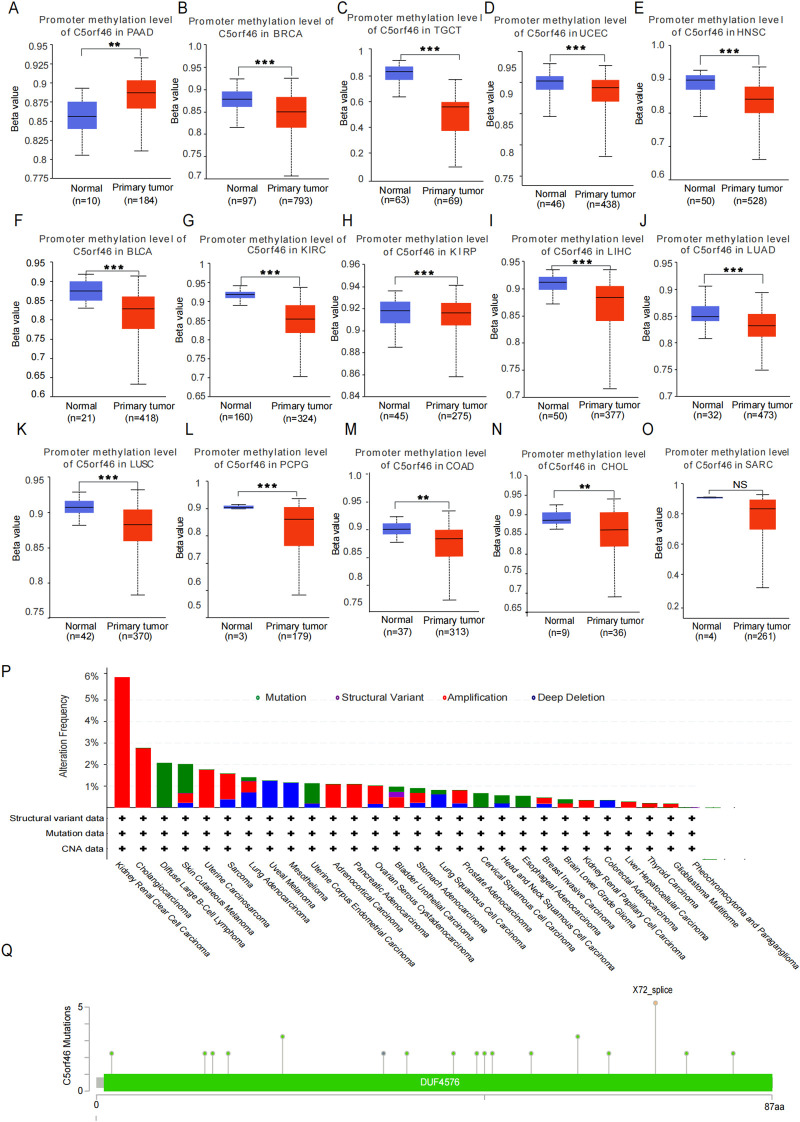
Promoter methylation of C5orf46 and the gene variations in cancers. The promoter methylation level C5orf46 gene in different types of cancers, including **(A)** PAAD, **(B)** BRCA, **(C)** TGCT, **(D)** UCEC, **(E)** HNSC, **(F)** BLCA, **(G)** KIRC, **(H)** KIRP, **(I)** LIHC, **(J)** LUAD, **(K)** LUSC, **(L)** PCPG, **(M)** COAD, **(N)** CHOL, and **(O)** SARC. **(P)** Mutation detection results pf C5orf46 gene in human cancers as well as **(Q)** the detailed detected mutation spots revealed by cBioPortal dataset (**p < 0.01, ***p < 0.001).

To explore the potential reason for the altered C5orf46 gene expression in human cancers, including KIRC, the methylation level of the gene was investigated based on UALCAN platform. The results revealed that, except for PAAD, which showed that C5orf46 gene methylation was higher in cancer compared to normal tissues, such trend was actually inconsistent with the lower gene expression in cancer; as for KIRC, as well as other cancers, for instance BRAC, LUAD, and LUSC, gene methylation was generally lower in cancer compared with the corresponding normal samples (**Figures 5A–N**). The results indicated that gene methylation accounts for at least part of the upregulated expression of C5orf46 in human cancers. Meanwhile, as for the mesenchymal-tissue-originated sarcomas, the gene methylation level was not statistically significantly different in tumor compared with normal tissues (**Figure 5O**), indicating a much more complex regulation mechanism in this kind of tumors.

### 3.6 Other genetic alterations of C5orf46 gene in cancers

Besides mRNA expression, to comprehensively the understand C5orf46 gene status in cancers, other genetic alterations, including gene mutation, protein structural variants, and copy number variation, were investigated based on the cBioPortal database. The results revealed that gene amplification was one of the main types of C5orf46 alteration in cancers; especially in KIRC, gene amplification has been the only type of alteration being detected so far (**Figure 5P**). Meanwhile, as for the other cancers, a certain percentage of deletion and genetic mutations, which were mostly single-nucleotide variations, was also discovered in several types of cancers, including skin cutaneous melanoma, diffuse large B-cell lymphoma, uterine corpus endometrioid carcinoma, cervical squamous cell carcinoma, head and neck squamous cell carcinoma, and esophageal adenocarcinoma (**Figure 5Q**).

### 3.7 C5orf46 centering protein–protein interaction network construction and functional enrichment analysis

To preliminarily explore the potential mechanism of C5orf46 regulation on KIRC, as well as other cancers development, STRING was used to construct a C5orf46-centered PPI network, followed by gene enrichment analysis, thus uncovering the probable signaling pathways in which C5orf46 and its interacting genes were involved. The results actually showed a consistent direction, aligning with the current understanding of C5orf46 as an exosome-containing gene that mainly participates in biological processes such as cell communication, secretion, and chemical synaptic transmission (**Supplementary Figures 2A–D**). The results supported further and deeper detailed exploration of the correlation between C5orf46 and the currently acknowledged exosome-related biological cellular effects.

### 3.8 C5orf46 related with TME angiogenesis

**Figure 6:**
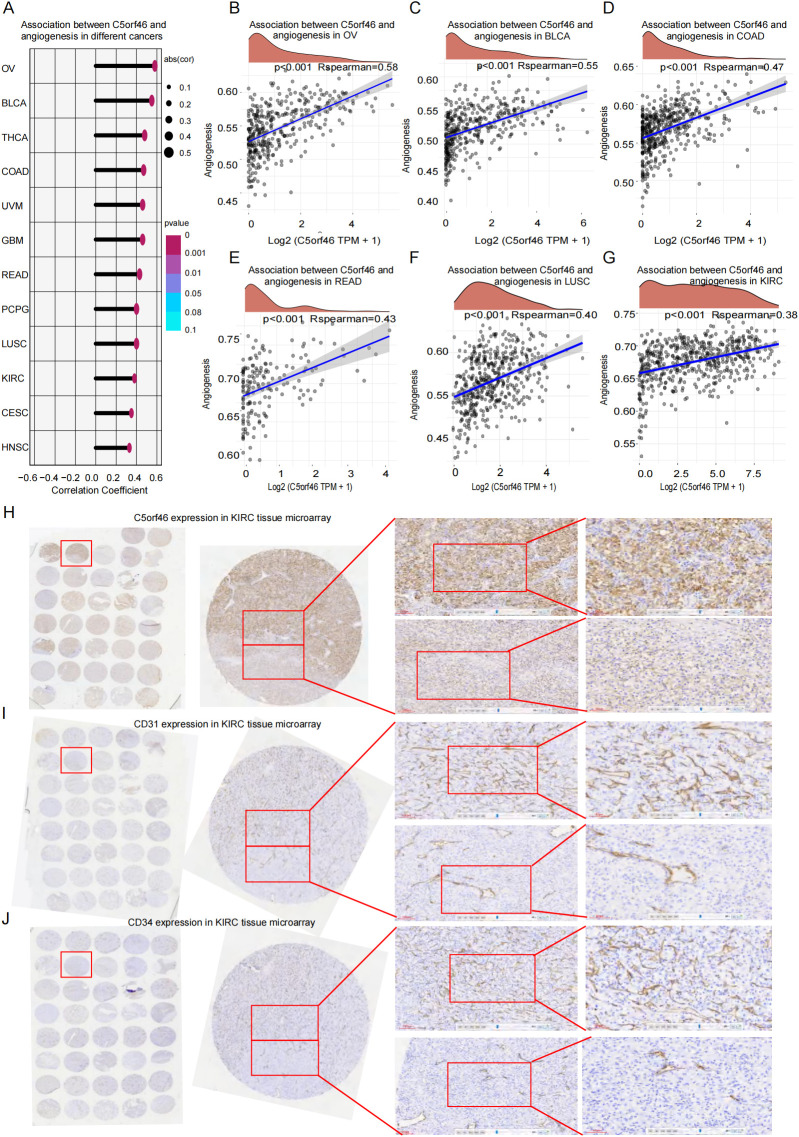
Association between C5orf46 gene and pan-cancer TME angiogenesis. **(A)** C5orf46 association with TME angiogenesis related genes signature which was calculated based on TCGA gene expression data in human cancers. C5orf46 association with TME angiogenesis related genes signature in individual cancers, including **(B)** OV, **(C)** BLCA, **(D)** COAD, **(E)** READ, **(F)** LUSC, and **(G)** KIRC (*R >*0.30 was considered correlated, *R* between 0.30 and 0.49 was considered preliminarily correlated, and *R* between 0.50 and 0.79 was moderately correlated. Meanwhile, *R >*0.80 was thought as strongly correlated). **(H)** A tissue microarray made of KIRC samples for IHC experiments detecting C5orf46 expression. The middle panel represents a tumor tissue from the left panel tissue microarray. The upper graphs (red box) in the right panel represents a KIRC cancer lesion with a higher C5orf46 expression, and the lower graphs (blue box) were a tissue lesion with a relatively lower expression (magnification: ×100 and ×200; the error bar represents 100 and 60 um in the left and right graph, respectively). **(I)** The tissue microarray is the same as above in the graph in **(F)** and IHC experiment detecting CD31 which is a classic gene marker for tissue angiogenesis. The middle panel represents the same tumor tissue as above—C5orf46 gene expression detection tissue with the same magnification and error bar representation. **(J)** The tissue microarray is the same as above in the graph in **(F)** and IHC experiment detecting CD34 which is another classic gene marker for tissue angiogenesis. The middle panel represents the same tumor tissue as above—C5orf46 gene expression detection tissue with the same magnification and error bar representation.

TME angiogenesis has been a major aspect during tumor development and metastasis. To validate the association between C5orf46 gene and tumor angiogenesis, we selected 32 angiogenesis-related genes and analyzed their association with C5orf46 expression in various cancers, and the results showed a statistically significant positive correlation between the gene and KIRC, as well as multiple other cancers, for instance, OV, BLCA, and COAD (**Figures 6A–G**).

Meanwhile, the IHC experiment that was conducted on KIRC tissue microarray also revealed that although C5orf46, CD31, and CD34 expressions vary in different cancer samples, the C5orf46 expression was indeed associated with both the number and area of CD31- and CD34-stained blood vessels (**Table 2**). We took a case with obvious heterogeneous C5orf46 expression as an example (**Figure 6H**). In the area with high C5orf46 expression, both the number of CD31-stained immature blood vessels (**Figure 6I**) and CD34-marked mature blood vessels (**Figure 6J**) were higher than in the area with a low C5orf46 expression, supporting the potential effect that C5orf46 has on TME angiogenesis. The colocalization of C5orf46 with CD31/CD34 has not been included in the study due to the reagent and equipment limitation in our laboratory, and also KIRC has been well known as a typical cancer rich in blood sinuses. Not only the number of blood vessels but also the vessel lumen structure, size, and morphology shall affect cancer development. More detailed experiments and analysis are still needed to further investigate the effect of C5orf46 on KIRC blood vessel remodeling.

**Table 2:** Correlation between C5orf46 expression and KIRC angiogenesis and T cell infiltration.

Parameters		C5orf46 expression		*T*-value	*P*-value
		Low expression	High expression		
CD31 stained blood vessels					
	Vessels number	39.5 (30.00, 56.25)	102.00 (886.00, 157.59)	-4.226	<0.001***
	Average area (pixels)	2,471 (1,790, 5,485)	1,641 (1,035, 3,257)	-2.192	0.028*
CD34 stained blood vessels					
	Vessels number	36 (17, 78)	94 (65, 146)	-3.092	0.002*
Average area (pixels)	2,069.50 (1148, 3,531)	1,716 (1,037, 1,967)	-1.706	0.088
CD8+ T cell infiltration		33.5 (28.50, 38.50)	36.00 (30.00, 40.05)	-0.860	0.394

*p < 0.05; ***p < 0.001.

### 3.9 C5orf46 correlates with ECM degradation and EMT transition in the metastasis process of cancers

Besides angiogenesis, ECM degradation has also been an important aspect of TME remodeling, which was not only a critical step for cancer metastasis but also a major biological process that reacts to oncogene-containing exosome regulation. To investigate the association between C5orf46 and ECM degradation, 23 ECM degradation-related genes were selected, and the analysis results revealed a significantly positive correlation in various cancers, including OV, BLCA, LUSC, and COAD (**Supplementary Figures 3B–J**); however, the association in KIRC was revealed to be not statistically significant (**Supplementary Figure 3A**).

Meanwhile, similar correlation trends were also observed between C5orf46 and cancer EMT transition. Although a positive correlation was revealed in various cancers between the C5orf46 gene and the signature composed of 14 EMT-related genes—for instance, in OV, BLCA, PAAD, and COAD (**Supplementary Figures 3L–Q**)—the correlation in KIRC was neither statistically significant (**Supplementary Figure 3K**). The significant correlation between the gene and multiple important cancer traits shall provide promising insights for further detailed investigation of the gene’s role in specific cancer progression.

### 3.10 Association between C5orf46 and cancer stemness index

**Figure 7:**
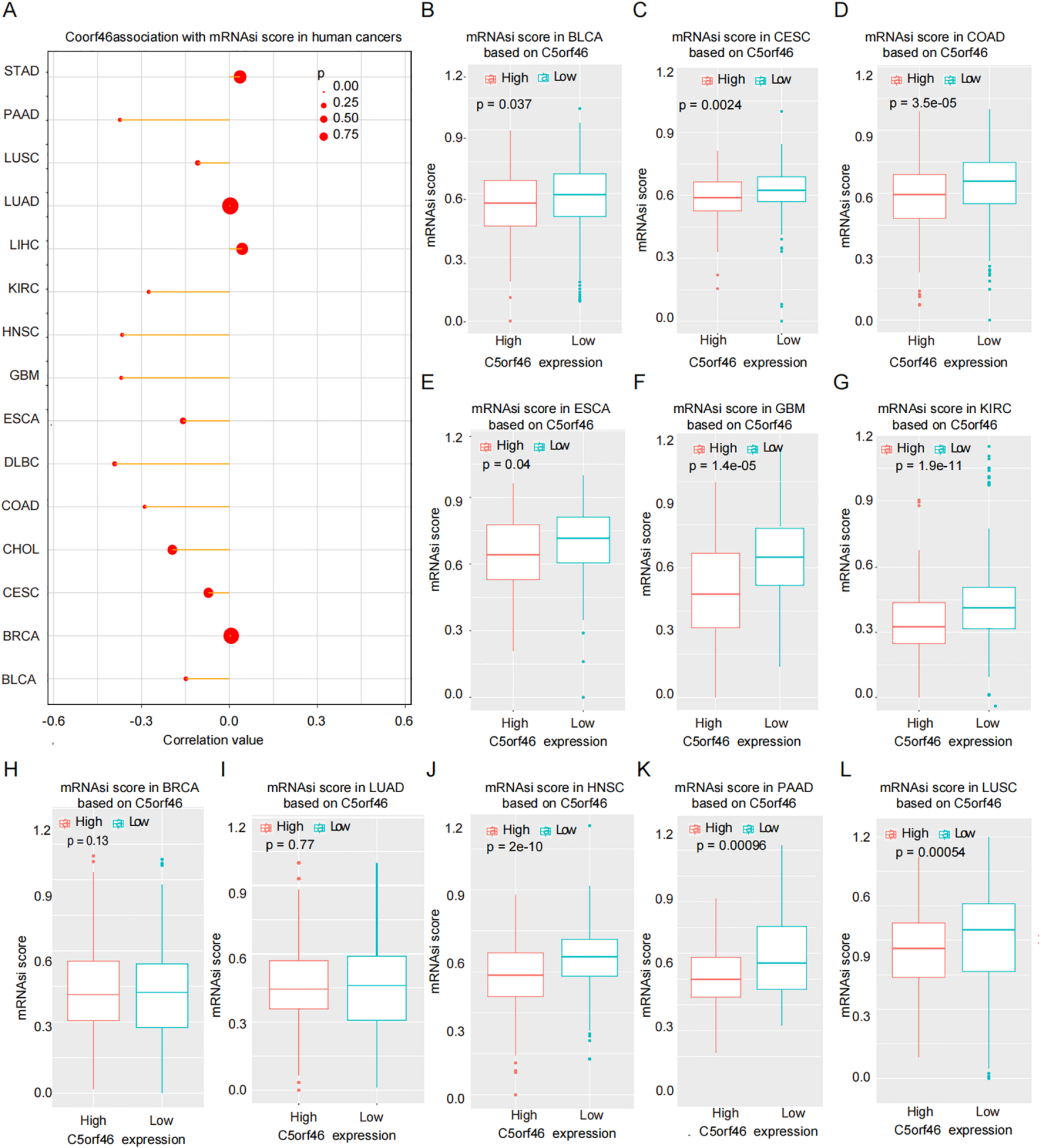
Cancer mRNAsi stemness score distribution in different C5orf46 gene expression samples. **(A)** C5orf46 association with mRNAsi score in human pan-cancers. Comparison of mRNAsi score in high-C5orf46 and low-expression groups in **(B)** BLCA, **(C)** CESC, **(D)** COAD, **(E)** ESCA, **(F)** GBM, **(G)** KIRC, **(H)** BRCA, **(I)** LUAD, **(J)** HNSC, **(K)** PAAD, and **(L)** LUSC. (*p < 0.05 was considered statistical significant).

Cancer stemness index is a recently developed indicator, which is calculated based on genes’ methylation and expression data, to evaluate the similarity between tumor cells and stem cells. In the study, we calculated the mRNAsi in different cancers based on TCGA pan-cancer genes’ expression data (**Figure 7A**), followed by analyzing its correlation with C5orf46 gene expression, and the results revealed that the mRNAsi index score differed significantly between high C5orf46 expression and the low-expression groups in KIRC, as well as various other cancers (**Figures 7B–L**). Interestingly, a similar trend was observed among various cancers, where the C5orf46 gene was negatively correlated with the mRNAsi index—namely, the index was higher in the low-C5orf46-expression groups, indicating a uniform effect of C5orf46 on cancer stemness.

### 3.11 Association between C5orf46 and HRR signature as well as MRR status analysis

**Figure 8:**
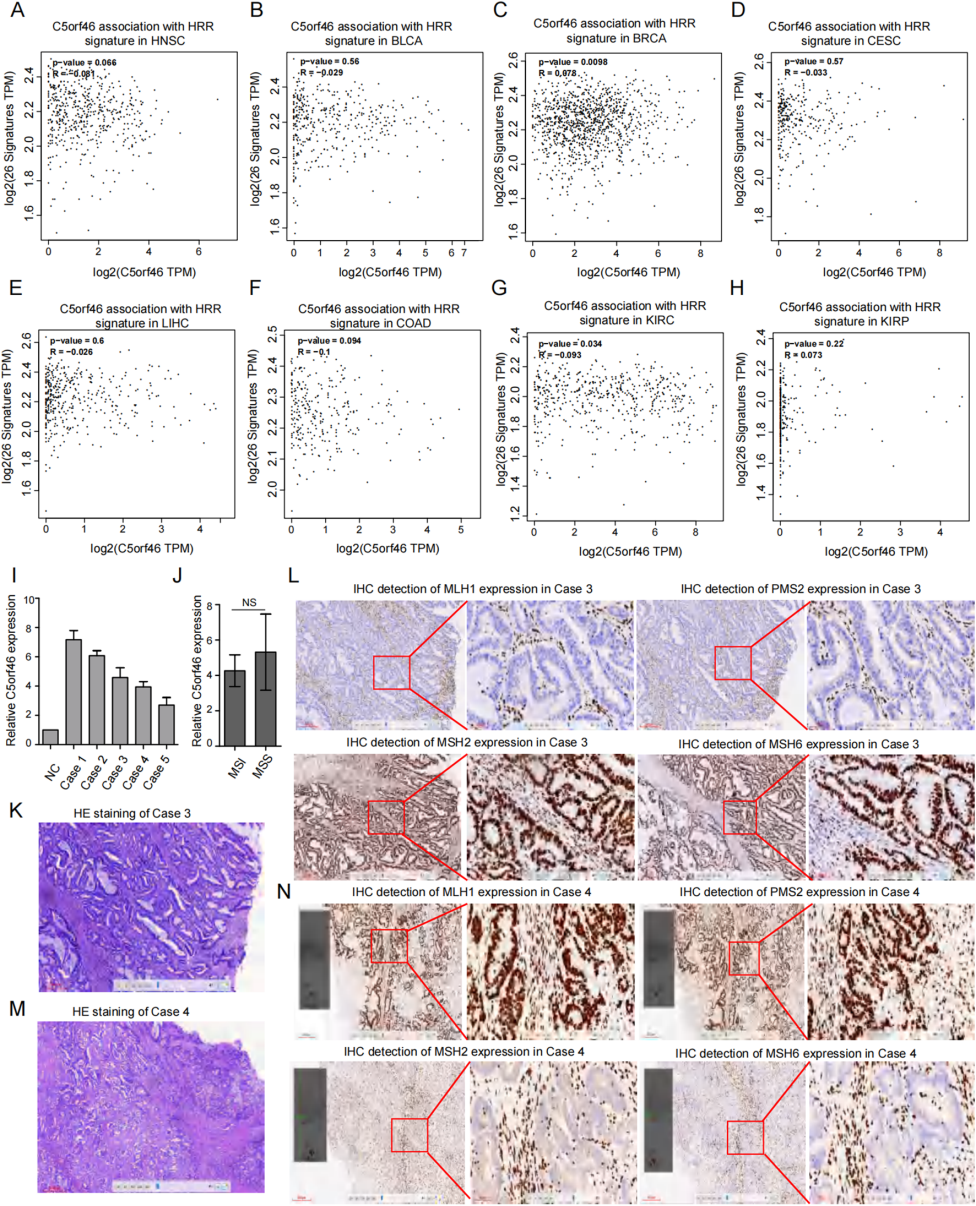
Association between C5orf46 and HRR related gene signature as well as MRR protein expression in pan-cancer. Association between C5orf46 gene and HRR related gene signature in **(A)** HNSC, **(B)** BLCA, **(C)** BRCA, **(D)** CESC, **(E)** LIHC, **(F)** COAD, **(G)** KIRC, and **(H)** KIRP. (*R >*0.30 was considered correlated, between 0.30 and 0.49 was considered preliminarily correlated, and between 0.50 and 0.79 was moderately correlated. Meanwhile, *R >*0.80 was thought as strongly correlated). **(I)** Real-time qRT-PCR detection of C5orf46 expression in five cases of COAD. **(J)** The five COAD cases were divided into MSS and MSI groups based on the IHC experiment of four MMR proteins, including MLH1, PMS2, MSH2, and MSH6 expression, and relative C5orf46 expression was compared between the two groups of samples (*p* < 0.05 was considered as statistically significant). **(K)** H&E staining and **(L)** IHC experiment of MMR proteins in case 3 which is one of the COAD samples that was detected as MSI. **(M)** H&E staining and **(N)** IHC experiment of MMR proteins in case 4, which is the other COAD sample that was detected as MSI.

Considering that DNA repair has been a major mechanism for maintaining cancer genome stability, which also contributes to the cancer stemness maintenance, we further analyzed the association between C5orf46 and 27 commonly known HRR genes, and the results revealed that only a mild interaction has been observed (**Figures 8A–H**), indicating that C5orf46 may not be a main regulator of DNA deficiency or HRR in cancers.

Besides HRD, given that MRR has also been another well-known DNA deficiency repair system, the association between C5orf46 expression and cancer MMR status was also preliminarily explored. In the study, we included five cases of COAD cancer samples, which is a common cancer type with a high dMMR ratio, and based on the IHC experiment of four MMR proteins (MLH1, PMS2, MSH2, and MSH6), two cases were revealed to be MSI. Meanwhile, the other three cases were MSS (**Figure 8I**). We compared the relative C5orf46 expression between the two MSI samples and three MSS cases, and no statistical significance was discovered (**Figure 8J**). Even between the two cases with MSI status, the MMR protein expressions were different—one showed loss of MLH1 and PMS2 expression (**Figures 8K, L**); meanwhile, the other showed loss of MSH2 and MSH6 expression (**Figures 8M, N**). Based on the current results, no specific correlation was found between C5orf46 and the cellular DNA deficiency repair system, although further and deeper analysis with a larger sample size covering more cancer types would help validate the conclusion.

A positive correlation was observed with specific cancer traits, including EMT, ECM degradation, and TME angiogenesis, while a negative correlation was found with cancer stemness index, and no specific correlation was observed with DNA repair, which may reflect the diversified functions of exosomal genes in cellular biological processes.

### 3.12 C5orf46 correlates with cancers’ immune landscape, especially cytotoxic T lymphocytes’ infiltration

**Figure 9:**
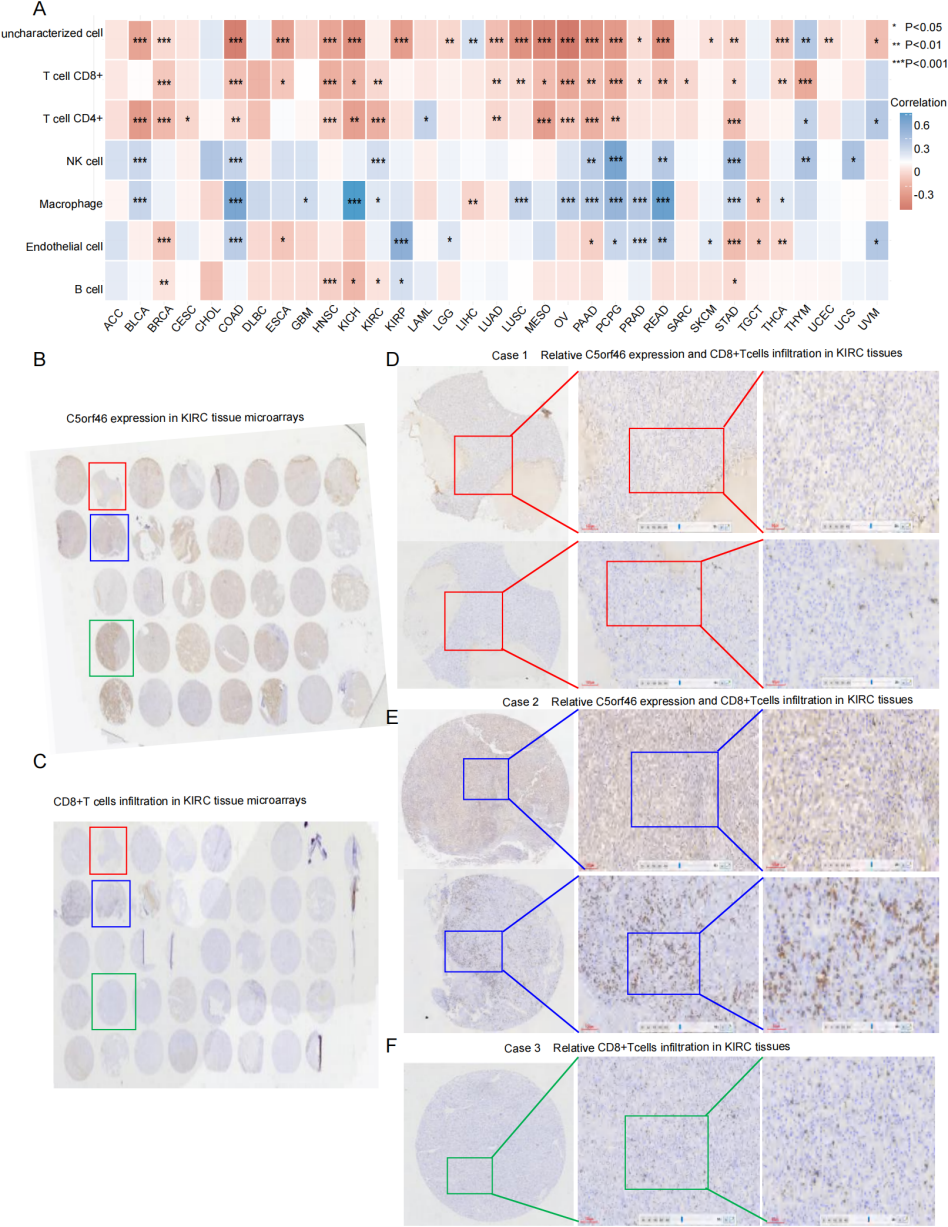
C5orf46 association with CD8+ T cells included TICs distribution in cancers. **(A)** Association between C5orf46 expression and various TICs distribution in pan-cancer. The same tissue microarray made of KIRC samples as in **Figure 6H** for IHC experiment showing the correlation between **(B)** relative C5orf46 gene expression and **(C)** CD8+ T cell infiltration—the red, blue, and green boxes in the graph represent three cases that were displayed in **(D–F)** graphs. **(D)** Relative C5orf46 expression (upper graph) and CD8+ T cell infiltration (lower graph) in the red boxed case. **(E)** Relative C5orf46 expression (upper graph) and CD8+ T cells’ infiltration (lower graph) in the blue boxed case. **(F)** Relative CD8+ T cell infiltration (lower graph) in the green boxed case, whose C5orf46 expression result was displayed in **Figure 6H** (magnification: ×100 and ×200; the error bar represents 100 and 60 um in the middle and right graph, respectively). *p<0.05, **p<0.01, and ***p<0.001.

To investigate the effect of the C5orf46 gene on cancer immune infiltration, the landscape of 22 tumor infiltration immunocytes (TIC) in different types of cancers was calculated based on the CIBERSORT database, followed by analysis of its relationship with C5orf46 expression. As indicated in the distribution heatmap, strong positive correlations between C5orf46 and macrophages were observed across multiple cancers, including BRCA, COAD, KICH, LUSC, OV, PAAD, READ, and so on. Meanwhile, negative correlations were observed with CD4+ T cell and CD8+ T cells in the abovementioned cancers. As for the association with endothelial cells, the distribution varied across different cancers, indicating a cancer-specific functional mode (**Figure 9A**).

Considering that CD8+ T cells are an important immune cell type with a significant cancer-killing function, the association between the gene and CD8+ T cell distribution was mainly validated using KIRC tissue microarray (the same as previously described, **Figures 9B, C**). What is controversial is that the association between the C5orf46 gene and CD8+ T cell distribution in KIRC was only stochastic. Although the level of CD8+ T cell infiltration varied in different samples, it seems inconsistent with the expression level of C5orf46 (**Figures 9D, E**). Especially in the same case with heterogeneous C5orf46 expression that was previously applied to observe angiogenesis, the CD8+ T cell infiltration was not statistically different in the areas with higher or lower C5orf46 expression (**Figure 9F**, **Table 2**).

Meanwhile, given that the dysfunction of cytotoxic T lymphocyte (CTL) is a major aspect during tumor immunosuppression, we also investigated the association of C5orf46 with CTL functions based on TIDE analysis. The analysis results revealed that the CTL dysfunction levels differed between high- and low-C5orf46-expression samples across multiple cancers, including lung cancer, melanoma, colorectal cancer, invasive breast cancer, and pancreatic cancer, but not in KIRC (**Supplementary Figures 4A–F**), indicating that C5orf46 indeed has potential value in predicting CTL function in cancers, but it functions in a cancer-specific manner, which means that it may only function in certain types of cancers. Further and deeper analysis with a larger sample size covering more cancer types would help validate the conclusion.

### 3.13 Tumor mutation burden, microsatellite instability, and immune therapy sensitivity analysis

TMB and MSI are not only well-acknowledged clinical indicators of the cancer immune microenvironment but also widely used biomarkers for predicting patients’ sensitivity to immunotherapy. Despite the significant correlation between C5orf46 and immune cell distribution, an analysis of the association with TMB and MSI revealed that C5orf46 only mildly affects MSI status in CHOL. As for the other cancers, no significant correlation was observed in either TMB or MSI analysis (**Supplementary Figures 4G, H**).

Meanwhile, consistent results have been revealed in the immunotherapy drug sensitivity analysis, which showed no statistically significant difference in C5orf46 expression between responders and non-responders after receiving immunotherapy drugs, including anti PD-1, PD-L1, and CTLA4 inhibitors (data not shown). The results indicated that, although C5orf46 relates with the immune infiltration landscape in cancers, it may not be a potential drug target for immunotherapy.

### 3.14 C5orf46 affects drug sensitivity in certain chemotherapies

**Figure 10:**
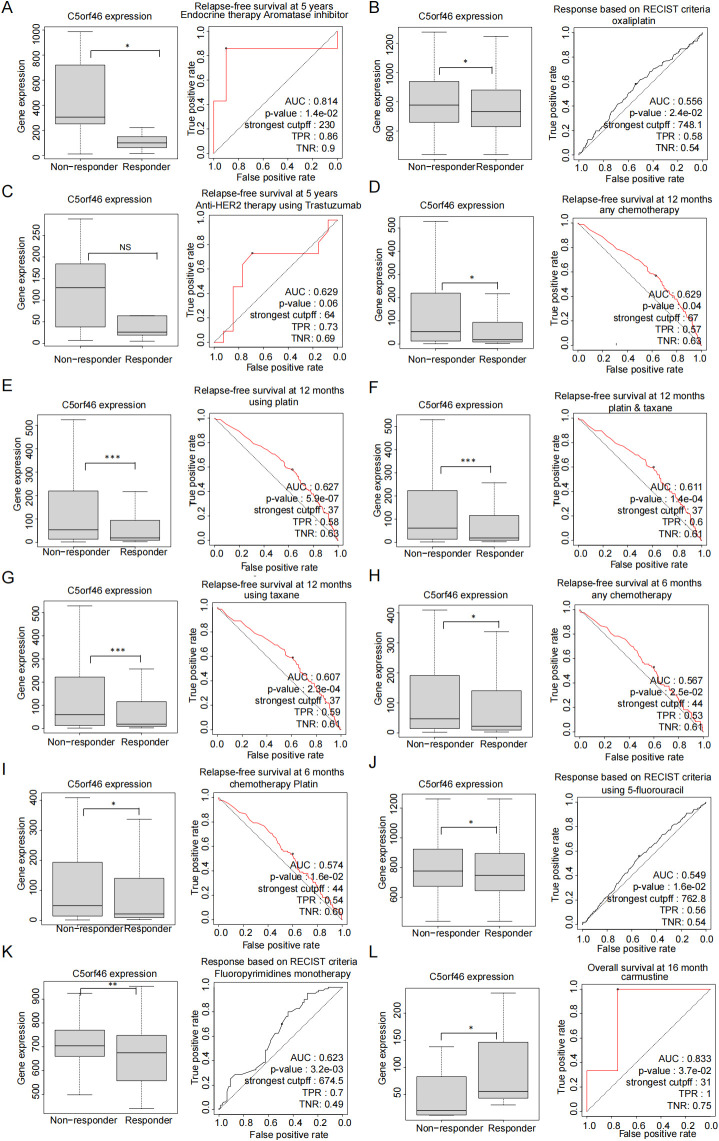
C5orf46 association with certain chemotherapy drugs’ sensitivity. C5orf46 gene expression difference between breast cancer responders and non-responders patients as well as the generated AUC curve after using **(A)** endocrine therapy aromatase and **(C)** anti-Her-2 trastuzumab. C5orf46 gene expression difference between colorectal cancer responder and non-responder patients as well as the generated ROC curve after using **(B)** chemotherapy oxaliplatin drugs. C5orf46 gene expression difference between ovary cancer responders and non-responder patients as well as the generated ROC curve after using **(D)** any chemotherapy drugs at 12 months, **(E)** platin at 12 months, **(F)** platin and taxane, **(G)** taxane, **(H)** any chemotherapy drugs at 6 months, and **(I)** platin at 6 months. C5orf46 gene expression difference between colorectal cancer responders and non-responder patients as well as the generated ROC curve after using **(J)** 5-fluorouracil and **(K)** fluoropyrimidines. C5orf46 gene expression difference between GBM cancer responders and non-responder patients as well as the generated ROC curve after using **(L)** camustine (*p < 0.05, **p < 0.01, ***p < 0.001).

Drug sensitivity is a critical and also insurmountable problem in clinical cancer treatment despite the lack of a specific correlation with immunotherapy drugs. To explore whether C5orf46 could be a potential indicator to predict chemotherapeutic responses in cancers, the ROCplotter database was accessed to evaluate the association between C5orf46 and therapeutic outcomes in certain cancer types. The results revealed that, in BRCA, C5orf46 expression was significantly higher in non-responders compared with responders after endocrine therapy using aromatase inhibitors as well as after anti-HER2 therapy using trastuzumab (**Figures 10A–D**). Similar trends were observed in ovarian and colorectal cancer patients, in whom C5orf46 expression was higher in non-responders compared with responders after chemotherapy using platin, taxane, 5-fluorouracil, oxaliplatin, and fluoropyrimidines (**Figures 10E–L**).

Additionally, based on the RNAactDrug platform, the top eight anti-C5orf46 small molecular compounds with FDR <0.05 were also revealed (**Table 3**). Although further in-depth experimental validation and clinical trials are needed before the clinical application of these drugs, the results shall provide promising insights for further research.

**Table 3:** The eight compounds correlated with C5orf46 based on RNAact drug platform.

Compounds	Omics	Source	Spearman.stat	Spearman.fdr
3-Bromo-4-n,n-bis-2’-cyanoethylaminobenzylidene rhodanine	Expression	CellMiner	0.453	0.030
Panobinostat	Expression	CCLE	0.400	7.643e-11
Sulfonaphtholazoresorcinol	Methylation	CellMiner	0.394	0.035
1,4-Dimethoxy-7-azaisoindole[2,1-a]quinoxalin-6(5h)-one	Methylation	CellMiner	0.374	0.042
1,3-Diphenyl-4-(3-phenyl-4,5-dihydro-1H-pyrazol-5-yl)-1	Expression	CellMiner	0.373	0.039
mercury(acetyloxy)(pentamethylphenyl)	Methylation	CellMiner	-0.390	0.018
Varacin trifluoroacetate salt	Expression	CellMiner	-0.398	0.045
8-Chloro-adenosine	Expression	CellMiner	-0.378	0.048

## 4 Discussion

Cancer has long been a major threat to human health, and carcinogenesis encompasses not only genetic alterations—for instance, oncogene activation and tumor suppressor gene mutation—but also extensive interactions between cancer cells and resident non-cancerous cells in the TME (33). The interactive patterns between the cancer cells and the surrounding TME components exert profound impacts on tumor progression, encompassing direct cell–cell contact and paracrine signaling. Among the paracrine mechanisms, the secretion of extracellular vesicles (EVs), particularly exosomes, constitutes a pivotal mode of cellular communication. Exosomes are increasingly recognized to execute diverse functions in carcinogenesis, including driving the malignant transformation of normal cells, participating in TME regulation, influencing the drug resistance of tumor cells, as well as regulating the pre-metastatic niche of tumors (34, 35).

In the study, we focused on a recently identified tumor regulatory gene, C5orf46. The gene was firstly exposed to us from previous studies when we analyzed the differentially expressed genes in primary cancers compared with corresponding normal control samples. The gene was generated repeatedly in multiple types of cancers—for instance, lung cancer (36), pancreatic cancer (37), and even osteosarcoma (38). This finding certainly sparked our interest, although we soon discovered that, up to now, only very limited functions have been reported about the gene. A pan-cancer analysis of the gene is anticipated to offer valuable guidance into elucidating its functions in the initiation and progression of diverse malignancies.

The study was started with an investigation of the fundamental physiochemical properties of C5orf46, the computed molecular weight, hydrophobicity/hydrophilicity feature as well as instability index which all supported C5orf46’s functions as a stable and hydrophilic cellular protein. Multiple databases support that the gene is mainly secreted and located in extracellular exosomes. However, the main limitation of the current validation is that while it is a potentially exosome-containing gene, exosomes have not been detected using electron microscopy because of equipment limitations in our laboratory. This part of the experimental validation has not been included in the study.

Following the understanding of the physiochemical property of C5orf46, the gene expression patterns as well as prognosis correlation were comprehensively investigated based on the TCGA pan-cancer data, which is a great resource for cancer-related gene analyses. While the TCGA-based analyses may harbor biases stemming from heterogeneous sample sources and processing protocols, they retain substantial clinical translational value, and multiple great discoveries have been found based on the data (39, 40). In the study, we encouragingly discovered that C5orf46 expression was widely upregulated in a broad spectrum of human cancers, especially in KIRC, which was indicated as the cancer type with the highest C5orf46 expression difference between cancer and normal tissues. To preliminary validate the altered expression of C5orf46 in KIRC compared with normal control samples, an IHC experiment using KIRC cancer samples from a local hospital was conducted, and the results supported that the gene is not only expressed at higher levels in cancers compared with corresponding normal tissues but is also associated with multiple cancer clinical features, including more advanced cancer stage and grade and metastatic status. Meanwhile, the higher gene expression was indicated to be statistically significantly correlated with worse patient OS and RFS across multiple cancer types.

To preliminarily explore the molecular basis underlying the upregulated expression of C5orf46 in cancers, its DNA methylation level was then evaluated, and we discovered that the methylation level of the Co5orf46 gene was lower in various cancers compared with corresponding normal control samples, except in PAAD. The results support that gene methylation may be a main regulator of the C5orf46 gene and accounted for at least part of the gene’s altered expression in human cancers. Beyond transcriptional dysregulation, other types of genetic alterations, including mutation ratio, protein structural variants, and copy number variations, that commonly affect gene functions were also analyzed, and the results indicated that although a certain percentage of deletion and single-nucleotide mutations were discovered, the gene amplification and protein gain of expression represent the predominant genetic alteration types of C5orf46 in human cancers.

Furthermore, to investigate the potential role of C5orf46 in cancers, a PPI network centered on the gene was constructed, followed by a preliminary analysis of the main enrichment of its interacting partner genes. Afterward, the associations between C5orf46 and multiple critical clinical cancer traits, including TME angiogenesis, ECM structure, tumor transition EMT, and immune modulation, were analyzed sequentially.

Angiogenesis has been a well-acknowledged driver of tumor growth and metastasis (41, 42), orchestrated by key factors including vascular endothelial growth factor (VEGF), VEGF receptor (VEGFR), fibroblast growth factor (FGF), platelet-derived growth factor (PDGF), and transforming growth factor β (TGF-β) (43–45). As a matter of fact, exosomes derived from cell lines or plasma sources of various human tumors—for instance, glioblastoma, pancreatic cancer, and nasopharyngeal carcinoma—have been reported to be able to induce angiogenesis *in vitro* and *in vivo* (46, 47). In the study, we discovered a significant positive correlation between exosomal C5orf46 and TME angiogenesis across multiple cancers, including COAD, CESC, KIRC, LUSC, LUAD, and READ. Notably, as KIRC is a highly vascularized malignancy characterized by abundant sinusoidal vessels, not only the number and area of blood vessels but also the vessel lumen structure and morphology affect cancer development. More detailed experiments are still needed to validate the association between C5orf46 and angiogenesis in KIRC.

In addition to angiogenesis, ECM degradation has also been a critical component of TME remodeling (48, 49). C5orf46 expression was significantly positively correlated with ECM degradation in multiple cancers, including KIRC, BLCA, BRCA, KIRP, PAAD, HNSC, CHOL, SKCM, THCA, and LIHC. Given that ECM degradation also facilitates cancer metastasis, to more comprehensively understand the potential effect of C5orf46 on cancer metastasis, we also analyzed the association between the gene and 29 cytoskeleton-dynamics-related genes that were related with actin filament stabilization, F-actin polymerization, and actin–myosin contractile force generation, but only a weak correlation was detected (data not shown).

Moreover, the EMT is also a pivotal process in cancer progression, which is defined by reduced E-cadherin expression, increased N-cadherin expression, and upregulation of regulators such as SNAL, proteases, and pluripotent transcription factors (50–52). In the study, we selected 14 genes that were well known to be related to the EMT, analyzed their correlation with C5orf46, and discovered a statistically significant positive correlation in multiple cancers, including BLCA, BRCA, CESC, CHOL, HNSC, COAD, KIRP, and LUSC, whereas no significant association was observed in KIRC. Meanwhile, as for the DNA deficiency and repair system, no specific correlation was observed between C5orf46 gene expression and HRR-related gene signatures in cancers.

Accumulating evidence demonstrate that exosomes mediate tumor immune evasion by transferring the bioactive cargos that they contain, such as proteins, mRNA, and miRNA, to certain receptor TME cells (53)—for instance, lung-cancer cell-derived exosomes have been reported to induce immune escape by reducing T cell activity, expressing PD-L1, and promoting tumor growth. Given the clinical significance of immunotherapy in cancer treatment, elucidating the regulatory mechanisms of tumor infiltration is imperative (54). In the study, the potential effect of C5orf46 on the cancer immune infiltration landscape was explored, and the results revealed that C5orf46 was strongly correlated with the distribution of multiple TICs, most notably macrophages, CD4+ T cells, and CD8+ T cells. While findings on KIRC remain controversial, differences in CTL dysfunction levels between high- and low-C5orf46-expression groups across cancers imply a regulatory role for C5orf46 in the tumor immune environment.

Furthermore, to evaluate the potential of C5orf46 as a therapeutic target, we explored the association between gene expression and therapeutic responses, and we discovered that although C5orf46 correlates with the TMB and MSI status in select cancers, no statistically significant difference in gene expression was observed between the responders and non-responders to anti-PD-1, anti-PD-L1, or anti-CTLA4 immunotherapies. Meanwhile, as for the chemotherapy drugs, C5orf46 expression significantly differed between chemoresistant and chemosensitive patients with BRCA, colorectal cancer, and GBM. Despite the inherent challenges in developing anti-exosome drugs (1) and the requirement for extensive *in vitro* validation and clinical trials prior to clinical translation, the results in the study shall provide promising perspectives for further clinical research into the oncogenic functions of C5orf46.

## Data Availability

The TCGA pan-cancer profiles used in the study can be downloaded from UCSC Xena (https://www.cancer.gov/ccg/research/genome-sequencing/tcga). The original contributions presented in the study are included in the article and **Supplementary Material**. Further inquiries can be directed to the corresponding authors.
