# Supplementary material for: C5orf46: a promising prognosis risk indicator with implication in the remodeling of KIRC and pan-cancer tumor microenvironments
Source: Front Oncol. 2026 May 4;16:1713635. doi: 10.3389/fonc.2026.1713635 (PMC13180555; doi:10.3389/fonc.2026.1713635)
Supplement: Supplementary file 1 [file DataSheet1.docx]

Supplementary Material

## Supplementary Figures


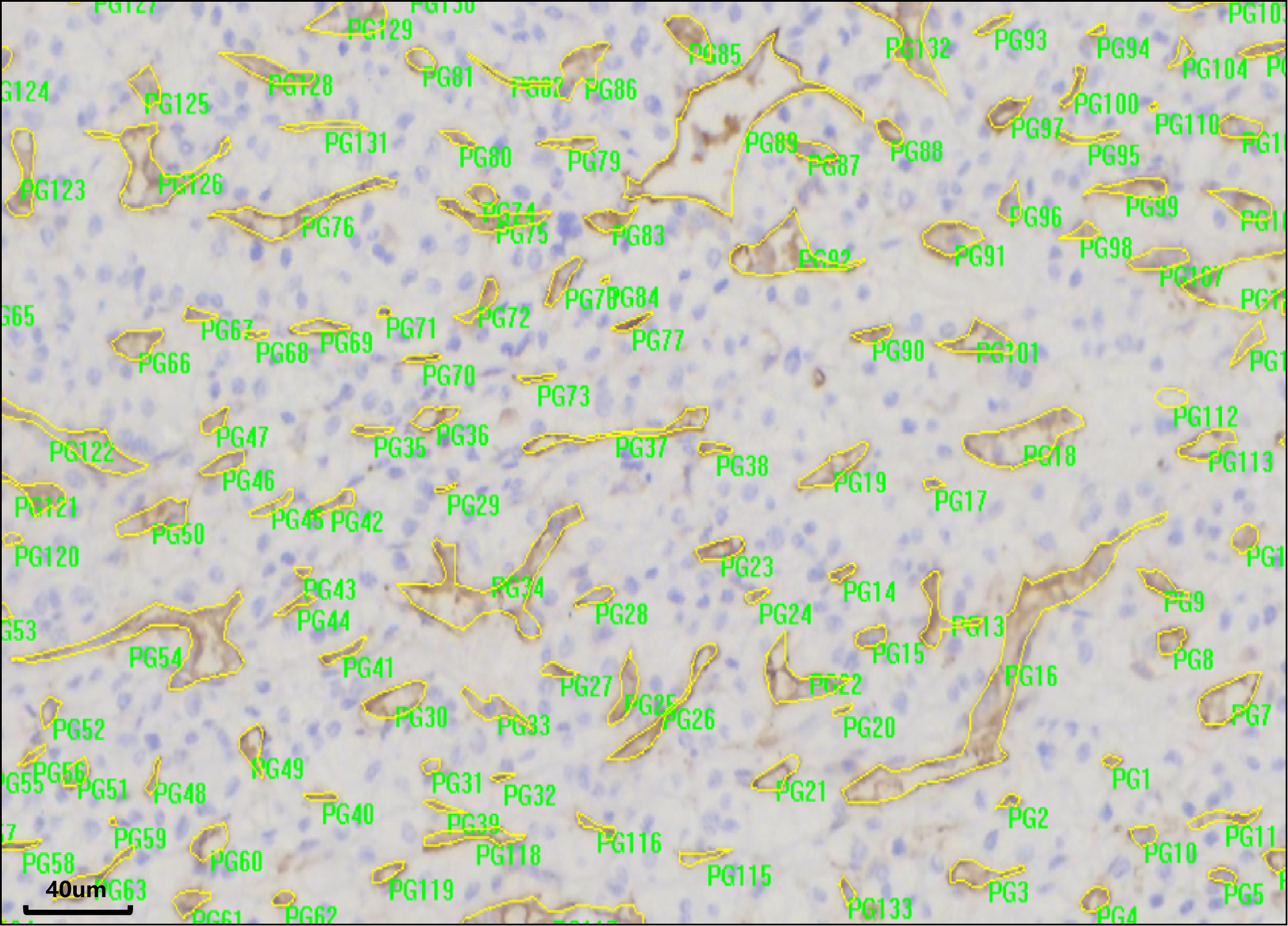


**Supplementary Figure 1.** A schematic diagram of microvessel density measurement in KIRC samples, the green marks in the figure indicate the tumor microvessels labeled by Image plus 6.0 analysis software.

**
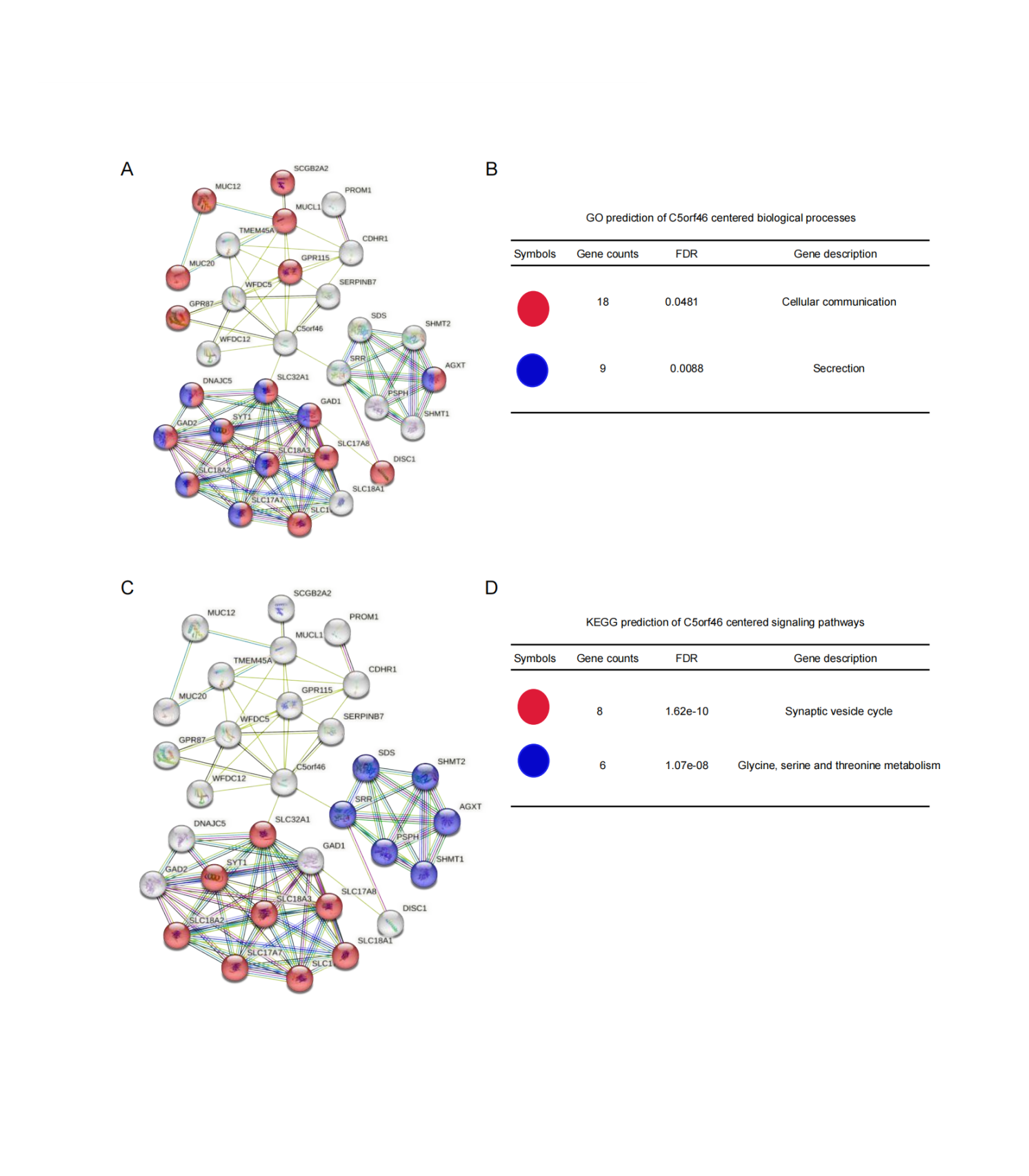
**

**Supplementary Figure 2.** **PPI network centering on C5orf46 gene and enrichment analysis**

1. The PPI network which is centered on C5orf46 gene for analyzing (B) the main biological functions C5orf46 and its connected genes mainly participated in.

(C)The PPI network centered on C5orf46 for analyzing (D) the main signaling pathways C5orf46 and its interacting genes involved in.

**
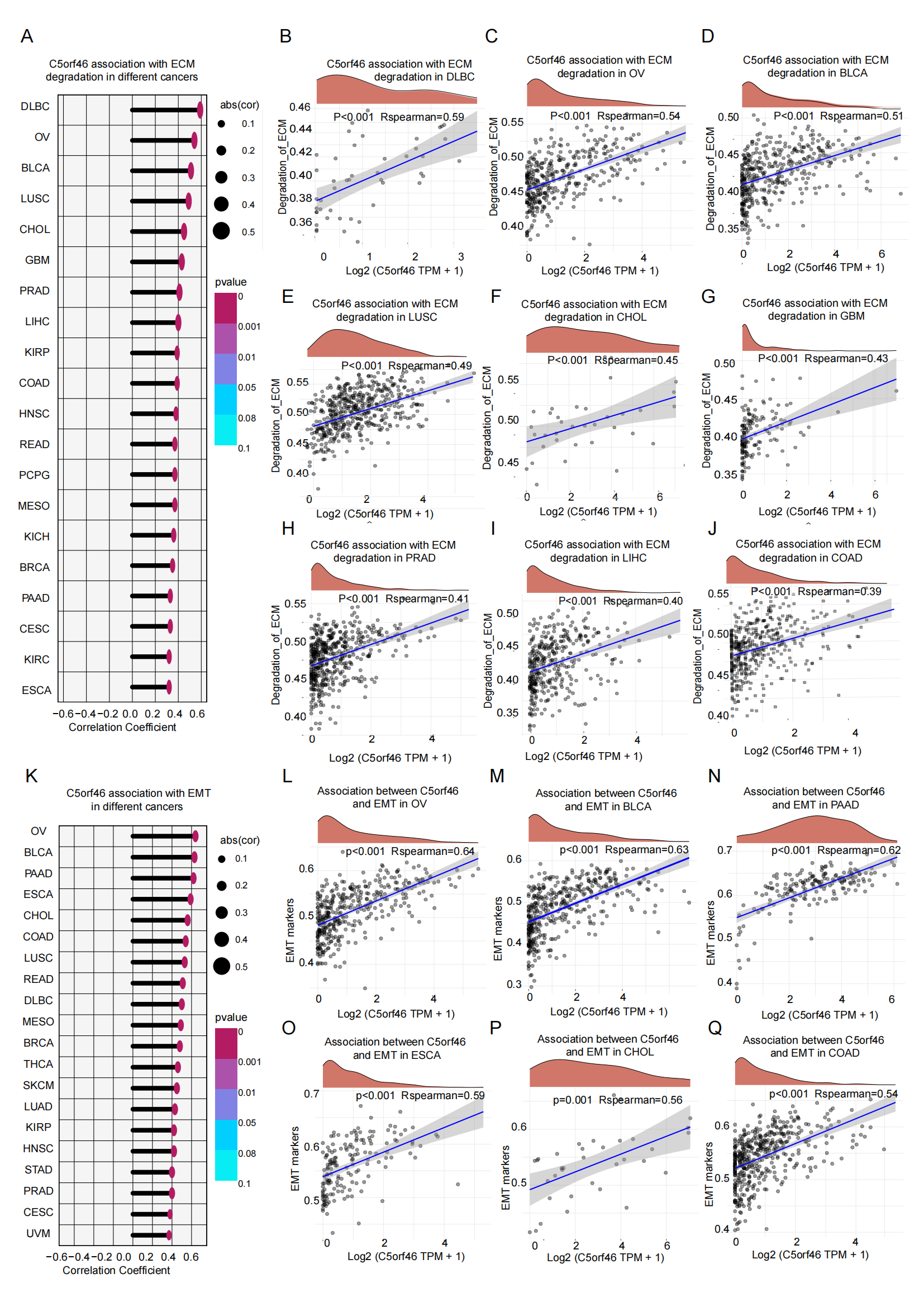
**

**Supplementary Figure 3.** **Association between C5orf46 and ECM degradation as well as EMT transition related gene signature in cancers**

1. C5orf46 association with ECM degradation which was calculated based on TCGA genes expression data in different human cancers. C5orf46 association with ECM degradation related gene signature in individual cancers, including in (B) DLBC, (C) OV, (D) BLCA, (E) LUSC, (F) CHOL, (G) GBM, (H) PRAD, (I) LIHC, (J) COAD. (K) C5orf46 association with EMT transition related gene signature that was calculated based on TCGA genes expression data in different human cancers. C5orf46 association with EMT transition related gene signature in (L) OV, (M) BLCA, (N) PAAD, (O) ESCA, (P) CHOL and (Q) COAD. (R>0.30 was considered correlated, R between 0.30~0.49 was considered preliminary correlated, and 0.50~0.79 was moderate correlated, meanwhile, R>0.80 was thought as strongly correlated).


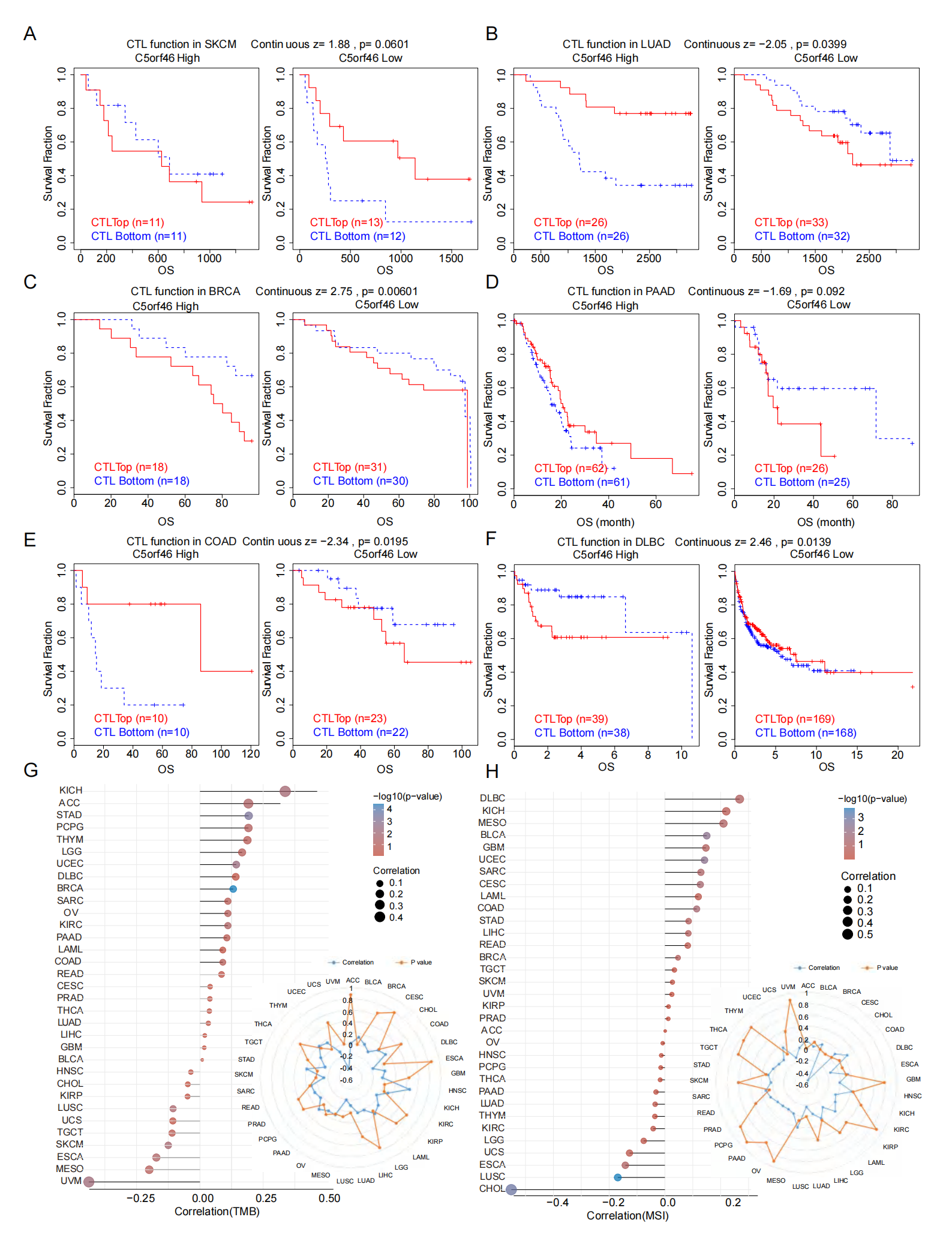


**Supplementary Figure 4.** **C5orf46 association with CTL dysfunction and MSI as well as TMB in human cancers**

Association between C5orf46 gene and CTL function status which was conducted based on TIDE platform in (A) SKCM, (B) LUAD, (C) BRCA, (D) PAAD, (E) COAD and (F) DLBC which diagrams revealed different CTLfunction in the patients with C5orf46 high and low expressions ( the statistical value was generated based on the platform with p<0.05 considered statistical significant). C5orf46 association with tumor (G) TMB and (H) MSI status in cancers which was conducted based on ACLBI platform.

**Supplementary Table 1.**  **The 32 TME angiogenesis related genes for evaluating the association with C5orf46**

| List of the 32 selected TME angiogenesis relating genes | | | | |
| --- | --- | --- | --- | --- |
| APOH | APP | CCND2 | CXCL6 | FGFR1 |
| FSTL1 | ITGAV | JAG1 | JAG2 | KCNJ8 |
| LPL | LRPAP1 | LUM | MSX1 | NRP1 |
| OLR1 | PDGFA | PF4 | PGLYRP1 | POSTN |
| PRG2 | PTK2B | SERPINA5 | STC1 | THBD |
| TIMP1 | TNFRSF21 | VAV2 | VCAN | VEGFA |
| VTN |  |  |  |  |

**Supplementary Table 2. The 23 selected ECM degradation related genes for evaluating the association with C5orf46**

| List of the 23 ECM degradation relating genes | | | | |
| --- | --- | --- | --- | --- |
| COL11A1 | COL10A1 | SFTPC | CTHRC1 | ABI3BP |
| PCOLCE2 | OGN | MMP1 | ADAMTS8 | CPB2 |
| GREM1 | SFTPA2 | CHRDL1 | WIF1 | CD36 |
| CXCL2 | CXCL13 | SFTPD | HHIP |  |
| MAMDC2 | LPL | IL6 | TNNC1 |  |

**Supplementary Table 3. The 14 EMT related genes for evaluating the association with C5orf46**

| List of the 14 EMT relating genes | | | | |
| --- | --- | --- | --- | --- |
| Vimentin | MMP9 | MMP2 | MMP3 | FOXC2 |
| TWIST1 | FN1 | ITGB6 | SNAI1 | SNAI2 |
| CDH2 | GSC | CDH1 | DSP |  |

**Supplementary Table 4. The 27 HRR related genes for evaluating the association with C5orf46**

| Detailed list of the 27 HRR relating genes | | | | |
| --- | --- | --- | --- | --- |
| ATM | BARD1 | BRCA1 | BRCA2 | BRIP1 |
| CDH1 | CDK12 | CHEK1 | CHEK2 | FANCA |
| FANCL | HDAC2 | PALB2 | PPP2R2A | PTEN |
| RAD51B | RAD51C | RAD51D | RAD54L | TP53 |
| ATRX | ADRID1A | BARD1 | BLM | MRE11 |

**Supplementary Table 5. Association between C5orf46 expression and KIRC clinical features in different patients gender**

| Parameters | | Female patient | | | Male patient | | |
| --- | --- | --- | --- | --- | --- | --- | --- |
|  |  | C5orf46 low | C5orf46 high | P value | C5orf46 low | C5orf46 high | P value |
| WHO/ISUP grade | |  |  |  |  |  |  |
|  | G1/G2 | 74(50.7) | 66(33.8) | 0.002** | 64(56.1) | 39(55.7) | 0.995 |
|  | G3/G4 | 72(49.3) | 129(66.2) |  | 50(43.9) | 31(44.3) |  |
| AJCC Stage | |  |  |  |  |  |  |
|  | Stage I | 84(56.4) | 77(39.9) | 0.013* | 75(64.7) | 31(43.0) | 0.018* |
|  | Stage II | 19(12.8) | 24(12.4) |  | 5(4.3) | 9(12.5) |  |
|  | Stage III | 26(17.4) | 54(28.0) |  | 22(19.0) | 21(29.2) |  |
|  | Stage IV | 20(13.4) | 38(19.7) |  | 14(12.0) | 11(15.3) |  |
| AJCC T Stage | |  |  |  |  |  |  |
|  | T1 | 84(56.4) | 80(40.8) | 0.005** | 75(64.7) | 34(47.2) | 0.013* |
|  | T2 | 21(14.1) | 31(15.8) |  | 6(5.1) | 11(15.3) |  |
|  | T3 | 38(25.5) | 82(41.9) |  | 35(30.2) | 25(34.7) |  |
|  | T4 | 6(4.0) | 3(1.5) |  | 0(0.0) | 2(2.8) |  |
| AJCC N Stage | |  |  |  |  |  |  |
|  | N0 | 71(94.7) | 72(90.0) | 0.277 | 54(98.2) | 43(93.5) | 0.227 |
|  | N1 | 4(5.3) | 8(10.0) |  | 1*(1.8) | 3(6.5) |  |
| AJCC M Stage | |  |  |  |  |  |  |
|  | M0 | 124(87.9) | 151(79.9) | 0.052 | 90(86.5) | 57(85.1) | 0.788 |
|  | M1 | 17(12.1) | 38(20.1) |  | 14(13.5) | 10(14.9) |  |

# *Represents p<0.05, **Represents p<0.01.
